# Tailoring a Functional Synthetic Microbial Community Alleviates *Fusobacterium nucleatum*‐infected Colorectal Cancer via Ecological Control

**DOI:** 10.1002/advs.202414232

**Published:** 2025-05-28

**Authors:** Zhongkun Zhou, Mengyue Yang, Hong Fang, Baizhuo Zhang, Yunhao Ma, Yongyuan Li, Yingjie Liu, Zeying Cheng, Yuanchun Zhao, Zhenzhen Si, Hongmei Zhu, Peng Chen

**Affiliations:** ^1^ School of Pharmacy Lanzhou University No. 199 Donggang West Road Lanzhou, Gansu 730000 P. R. China

**Keywords:** colorectal cancer, fusobacterium nucleatum, lipid metabolism, metabolic network reconstruction, synthetic microbial community

## Abstract

Polymorphic microbiomes play important roles in colorectal cancer (CRC) occurrence and development. In particular, *Fusobacterium nucleatum* (*F. nucleatum*) is prevalent in patients with CRC, and eliminating it is beneficial for treatment. Here, multiple metagenomic sequencing cohorts are combined with multiomics to analyze the microbiome and related functional alterations. Furthermore, local human metagenome and metabolomics are used to discover commensal consortia. A synthetic microbial community (SynCom) is then designed by metabolic network reconstruction, and its performance is validated using coculture experiments and an AOM‐DSS induced mouse CRC model. The sequencing result shows that *F. nucleatum* is more abundant in both the feces and tumor tissues of CRC patients. It causes alterations through various pathways, including microbial dysbiosis, lipid metabolism, amino acid metabolism, and bile acid metabolism disorders. The designed SynCom contains seven species with low competition interrelationship. Furthermore, the SynCom successfully inhibits *F. nucleatum* growth in vitro and achieves colonization in vivo. Additionally, it promotes *F. nucleatum* decolonization, and enhances tryptophan metabolism and secondary bile acid conversion, leading to reduced lipid accumulation, decreased inflammatory reaction, and enhanced tumor inhibition effect. Overall, the bottom‐up designed SynCom is a controllable and promising approach for treating *F. nucleatum*‐positive CRC.

## Graphical abstract

The bottom‐up strategy based on multiomics data is used for the SynCom design, and it successfully inhibited *F. nucleatum* growth and achieved stable colonization in vivo. In addition, it promoted tryptophan metabolism and secondary bile acid conversion, reduced lipid accumulation, relieved microbiome disorder, decreased inflammatory reaction, and enhanced tumor inhibition effect.

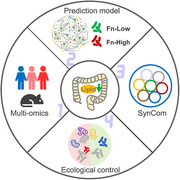

## 1 Introduction

Colorectal cancer (CRC) accounts for approximately one in ten cancer‐related deaths, and its etiology is multifactorial.^[^ ^1^ ^]^ Recently, a polymorphic microbiome was acknowledged as a new cancer hallmark, highlighting its important roles.^[^ ^2^ ^]^ Over the last decades, several bacteria, such as *Fusobacterium nucleatum* (*F. nucleatum*), bft‐producing *Bacteroides fragilis* (*B. fragilis*), pks+ *Escherichia coli* (*E. coli*), have been confirmed as harmful in the occurrence, development, metastasis, and chemoresistance of CRC.^[^ ^3^ ^]^ In particular, *F. nucleatum* has attracted much attention for its prevalence and carcinogenicity, and *F. nucleatum*‐positive tumors have benefited from anti‐fusobacterial therapy.^[^ ^4^ ^]^

Although metronidazole was demonstrated as efficient in slowing *F. nucleatum*‐infected tumor growth, such broad‐spectrum antibiotics can induce gut microbiota dysbiosis.^[^ ^5^ ^]^ Several more precise strategies have been proposed. First, to avoid the harmful effects of broad‐spectrum antibiotics, narrow‐spectrum treatment strategies were developed and phage‐guided modulation was found to be effective. Irinotecan‐loaded dextran nanoparticles covalently linked with azide‐modified phages and silver nanoparticles assembled with M13 phage achieved *F. nucleatum*‐targeted clearance.^[^ ^6^, ^7^ ^]^ Second, some beneficial microorganisms, including *Streptococcus salivarius* and *Saccharomyces cerevisiae* JKSP39, can alleviate *F. nucleatum*‐induced pathogenicity in a narrow‐spectrum approach.^[^ ^8^, ^9^ ^]^ Third, compared with biotherapy, chemotherapy is more widely applied in clinic. Therefore, exploring chemical drugs through drug rediscovery and novel compound synthesis has been undertaken in recent years. For instance, nitisinone was identified as a new *F. nucleatum* selective inhibitor.^[^ ^10^ ^]^ Higenamine derivatives obtained an MIC_50_ of 0.005 µM without toxicity in intestinal bacteria.^[^ ^11^ ^]^ Fourth, based on chemical drugs, optimization of drug formulation can produce better treatment effects. Nanomedicines, such as the *F. nucleatum* cytoplasmic membrane fused with colistin‐loaded liposomes, controllable supramolecular nanoparticle, and OLP/PP nanoassembly, also achieved selectively *F. nucleatum* inhibition.^[^ ^12^, ^13^, ^14^ ^]^ However, the influence of *F. nucleatum* on CRC development involves multiple aspects, such as the dysbiosis of gut microbiome, metabolic disorders and so on. Therefore, simply eliminating *F. nucleatum* may result in limited benefits, indicating the need for systematic regulation.

As one systematic biological therapy, fecal microbial transplantation (FMT) is applied in recurrent *Clostridium difficile* (*C. difficile*) infection and has been approved by the Food and Drug Administration (FDA) in 2013. Recently, SER‐109, composed of purified Firmicutes spores, led to less frequent *C. difficile* recurrence and was also approved by the FDA, highlighting the significance of microbiome therapy.^[^ ^15^ ^]^ Previous studies demonstrated that a higher community diversity could result in better colonization resistance against bacterial pathogens.^[^ ^16^ ^]^ Therefore, the design and use of defined microbial communities to modulate the microbial and metabolic disorders is a more sustainable choice.

Synthetic microbial communities (SynComs) refer to a reasonable assembly of microorganisms, designed to include one or more specific functions. SynComs have been widely used in agriculture, food industry, medical industry and so on.^[^ ^17^ ^]^ In humans, gut microbial communities have many beneficial functions, such as co‐metabolism, fermentation, eco‐resilience, and immune training. These microbes represent a robust ecosystem to protect host from pathogen invasion and colonization. Hence, therapy based on SynComs is attractive and promising. With the expanding culturing capacity and sequencing data, a top‐down approach (beginning with isolation and culture) and a bottom‐up strategy (using multiomics data and computational model construction) has resulted in rapid advances.^[^ ^18^, ^19^ ^]^ Faciliated by culturomics, a complex synthetic community (hCom2) containing 119 bacteria exhibited robust colonization resistance against pathogenic *E. coli*.^[^ ^20^ ^]^ Nevertheless, such complex microbiota are inconvenient for cultivation, storage, and quantification. Thus, some researchers tried to construct a SynCom with minimal members while retaining specific functions. By iteratively constructing smaller SymComs, Honda et al. identified a 17‐strain SynCom to induce Treg cells,^[^ ^21^ ^]^ and further isolated a consortium of 11 bacterial strains from feces to enhance host resistance against *Listeria monocytogenes* infection and anti‐cancer immunity.^[^ ^22^ ^]^ Recently, Kim et al. isolated a commensal bacterial consortium from feces, and these 18 commensal strains effectively decolonized Enterobacteriaceae and alleviated intestinal inflammation.^[^ ^23^ ^]^ However, such an iterative process is inefficient and labor‐intensive. The bottom‐up strategy focuses on assessing the function of individual species, followed by assembling SynComs to achieve specific functions. Usually, identification of these microbial targets is dependent on omics‐driven analyses. Furthermore, network‐based and correlation analyses are necessary to predict the bacterial or host‐bacteria interactions. Recently, Almeida et al. used a large‐scale dataset of 12,238 public human gut metagenomes and machine learning analyses, and identified a gut microbiome signature associated with Enterobacteriaceae colonization status.^[^ ^24^ ^]^ Daniel and his colleagues reanalyzed 16 S rRNA, metagenome and genomes to design GUT‐103 (17 strains) and GUT‐108 (11 strains), which reversed chronic immune‐mediated colitis and restored intestinal homeostasis.^[^ ^18^ ^]^ Moreover, various computational methods have been developed to facilitate the selection and optimization of SynComs. For predicting the dynamics of SynComs, a generalized Lotka–Volterra model was widely used.^[^ ^25^ ^]^ Regarding species’ interaction modeling, genome‐scale metabolic networks construction, including Pathway Tools,^[^ ^26^ ^]^ CarveMe,^[^ ^27^ ^]^ Kbase,^[^ ^28^ ^]^ and MiSCoTo,^[^ ^29^ ^]^ were developed to faciliate the minimal synthetic community design.

In this study, multiomics data, including the metagenome, metabolomics, transcriptome, methylome and proteome, were integrated to reveal functional and microbial disorders correlated with *F. nucleatum*‐infected CRC patients. Based on dysbiosis, identification a consortium to regulate these “alterations” is essential. Therefore, machine learning and correlation analyses were used to predict the potential microbial consortium for resisting *F. nucleatum*. Furthermore, genomic metabolic network reconstruction was performed to obtain the minimal microbial community while retaining specific functions. Finally, a SynCom containing 7 bacteria was designed, and the ability of the SynCom to eliminate *F. nucleatum* and alleviate *F. nucleatum*‐infected CRC was verified in vitro and in vivo to present a promising biotherapy for CRC patients.

## 2 Results

### 2.1 *F. nucleatum* is Enriched in CRC Patients and Correlated with Lipid Metabolism Disorder

**Figure 1:**
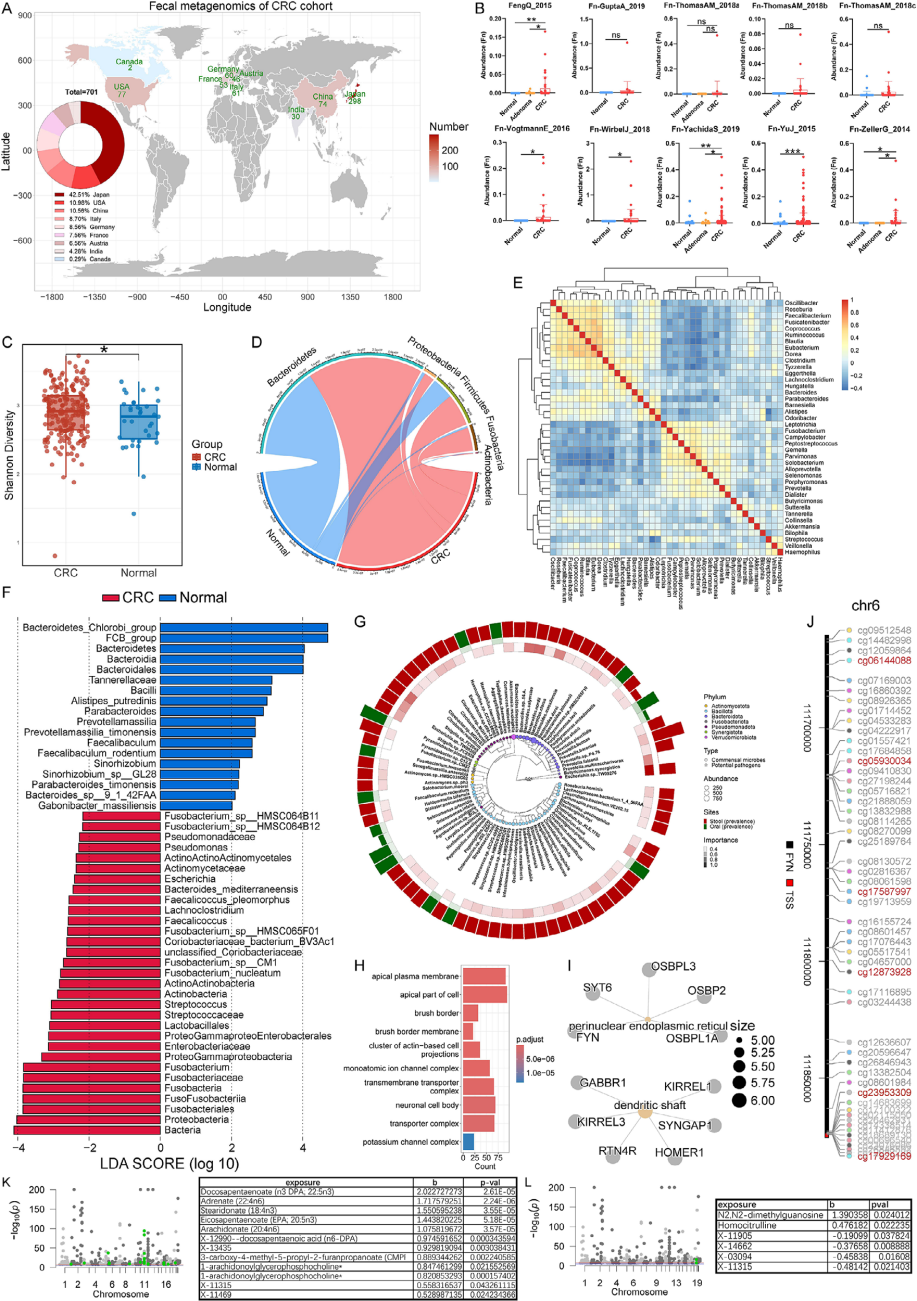
*F. nucleatum* is enriched in CRC patients and correlated with lipid metabolism disorder. Geographical distribution of the fecal metagenomic sequencing cohorts of CRC patients A); Shannon diversity between normal and CRC groups. *P < 0.05 by Student's t‐test B); the most abundant five phyla in different groups (the width of the line is directly proportional to the abundance of the microbe) C); the abundance of Fn in different cohorts of curatedMetagenomicData. Data are mean ± SEM. *P < 0.05, **P < 0.01, ***P < 0.001 by Student's t‐test D); SparCC correlation analysis of the most abundant 40 genera E); LEfSe analysis between healthy and CRC groups F); tumor microbiome used for prognosis predication (the third circle represents the regression coefficient b of the Cox proportional hazards model; the potential pathogens were species that were reported to be enriched in CRC patients; the oral bacteria were species that belong to the Oral Taxon in Human Oral Microbiome Database) G); GO enrichment analysis of genes that are positively correlated with Fn (*P* < 0.05 by t‐test of Pearson correlation) H); GO enrichment analysis of methylated genes that are positively correlated with Fn (*P* < 0.05 by t‐test of Pearson correlation) I); the distribution of methylated sites positively correlated with Fn in *FYN* gene (*P* < 0.01 by t‐test of Pearson correlation) J); Mendelian randomization analysis between CRC and blood metabolomics. The green points represent statistically significant SNPs K); Mendelian randomization analysis between *F. nucleatum* and blood metabolomics. The green points represent statistically significant SNPs L).

Fecal metagenomic sequencing data from eleven cohorts were downloaded from curatedMetagenomicData, which included 701 CRC patients, 142 adenoma patients, and 700 healthy individuals (**Figure** **1**A). The abundance of *F. nucleatum* (Fn) in feces was higher in CRC patients than in adenoma patients and healthy individuals (Figure 1B). Based on the KEGG annotations of metagenome data, the upregulated pathways of Fn (*P* < 0.05) included inosine, adenosine, rhamnose biosynthesis and pyruvate fermentation (Figure S1A, Supporting Information). The tissue microbiome data were downloaded from The Cancer Microbiome Atlas (TCMA), including 35 healthy samples and 217 CRC samples. In terms of the tissue microbiome, the alpha diversity was increased in CRC patients compared with that in healthy individuals (Figure 1C). Samples from these two groups were separated based on multidimensional scaling analysis (Figure S1B, Supporting Information). The CRC group contained more Proteobacteria and Fusobacteria, whereas the normal group contained more Firmicutes (Figure 1D). Then, the phylogenetic tree of the top 40 genera was constructed, which showed that Fusobacteria contained two members (*Fusobacterium* and *Leptotrichia*) (Figure S1C, Supporting Information). The correlation calculated by SparCC showed two distinct clusters. The healthy cluster contained more *Roseburia*, *Faecalibacterium*, *Eubacterium*, *Dorea*, *Clostridium*, *Bacteroides*, *Parabacteroides* and *Alistipes*, whereas the CRC cluster contained more *Leptotrichia*, *Fusobacterium*, *Campylobacter*, *Peptostreptococcus*, *Gemella*, *Parvimonas*, *Solobacterium*, *Alloprevotella*, *Selenomonas*, *Porphyromonas*, *Prevotella*, and *Dialister* (Figure 1E). LEfSe analysis demonstrated that *Fusobacterium nucleatum*, *Fusobacterium* sp. CM1, *Fusobacterium* sp. HMSC065F01, *Fusobacterium* sp. HMSC064B11 and *Fusobacterium* sp. HMSC064B12 were enriched in the CRC group (Figure 1F). Furthermore, among the eighteen species in *Fusobacterium*, most were increased in CRC patients (Figure S1D, Supporting Information).

Next, we analyzed the tumor microbiome combined with multiomics data of CRC patients from TCGA. First, patients were classified into alive and deceased, and the random forest model based on AUCRF package selected 32 markers with the highest prediction accuracy, including *A. muciniphila*, Fusobacteriia, *Bacteroidetes* and so on (Figure S1E, Supporting Information). Moreover, *B. fragilis*‐negative patients had a better prognosis than that of *B. fragilis*‐high patients (Figure S1F, Supporting Information). Second, multiple machine learning models were used to predict patient survival time, with the random forest+plsRCox achieving the best prediction performance (Figure S1G, Supporting Information). Next, the top 80 prognostic markers were used for phylogenetic tree construction, many of which belonged to oral microorganisms (Figure 1G). After matching the TCMA and TCGA databases, both RNA‐Seq and tumor microbiome data were available for 62 CRC samples. Pearson correlation analysis showed that 1172 genes were negatively correlated with Fn, and 1536 genes were positively correlated with Fn (*P* < 0.05). Enrichment analysis based on these genes indicated that Fn may promote the expression of intestinal cell transporters, apical plasma membrane, and ion channel complex genes (Figure 1H). Next, we analyzed 485,577 methylation sites, and 2373 sites were significantly correlated with Fn (*P* < 0.01). Enrichment analysis based on these methylation sites indicated that Fn may promote demethylation of protooncogenes (*FYN*), oxysterol (*OSBPL1A*, *OSBP2*, *OSBPL3*), and phospholipid (*SYT6*) metabolism genes (Figure 1I,J). Next, the RPPA protein expression dataset was used to identify Fn‐related proteins (*P* < 0.05), and proteins positively correlated with Fn were enriched in apical plasma membrane, Bcl‐2 family protein and so on. (Figure S1H, Supporting Information). Finally, the somatic mutation dataset was analyzed. Single nucleotide polymorphism (SNP) sites positively correlated with *Fusobacterium* were mainly annotated to the *TTC39A* gene, which was reported to promote breast cancer tumorigenicity.^[^ ^30^ ^]^

To verify the relationship between Fn and CRC, mendelian randomization (MR) analysis was performed. The results demonstrated that Fn was positively correlated with CRC (se = 0.24, *P* = 0.004, Figure S1I, Supporting Information). Moreover, MR analysis between blood metabolomics and CRC revealed that the metabolites positively correlated with CRC were mainly lipid molecules (Figure 1K). MR analysis between blood metabolomics and Fn revealed that the metabolites positively correlated with Fn included N2,N2‐dimethylguanosine and homocitrulline, which are correlated with glycolysis and arginine synthesis (Figure 1L).

By integrating the fecal metagenome, tumor microbiome, and multiomics data, we demonstrated that enrichment of *F. nucleatum* in CRC patients was correlated with metabolic dysfunction, such as lipid metabolism.

### 2.2 Prediction of Commensal Consortia for *F. nucleatum* Decolonization and their Potential Functions

**Figure 2:**
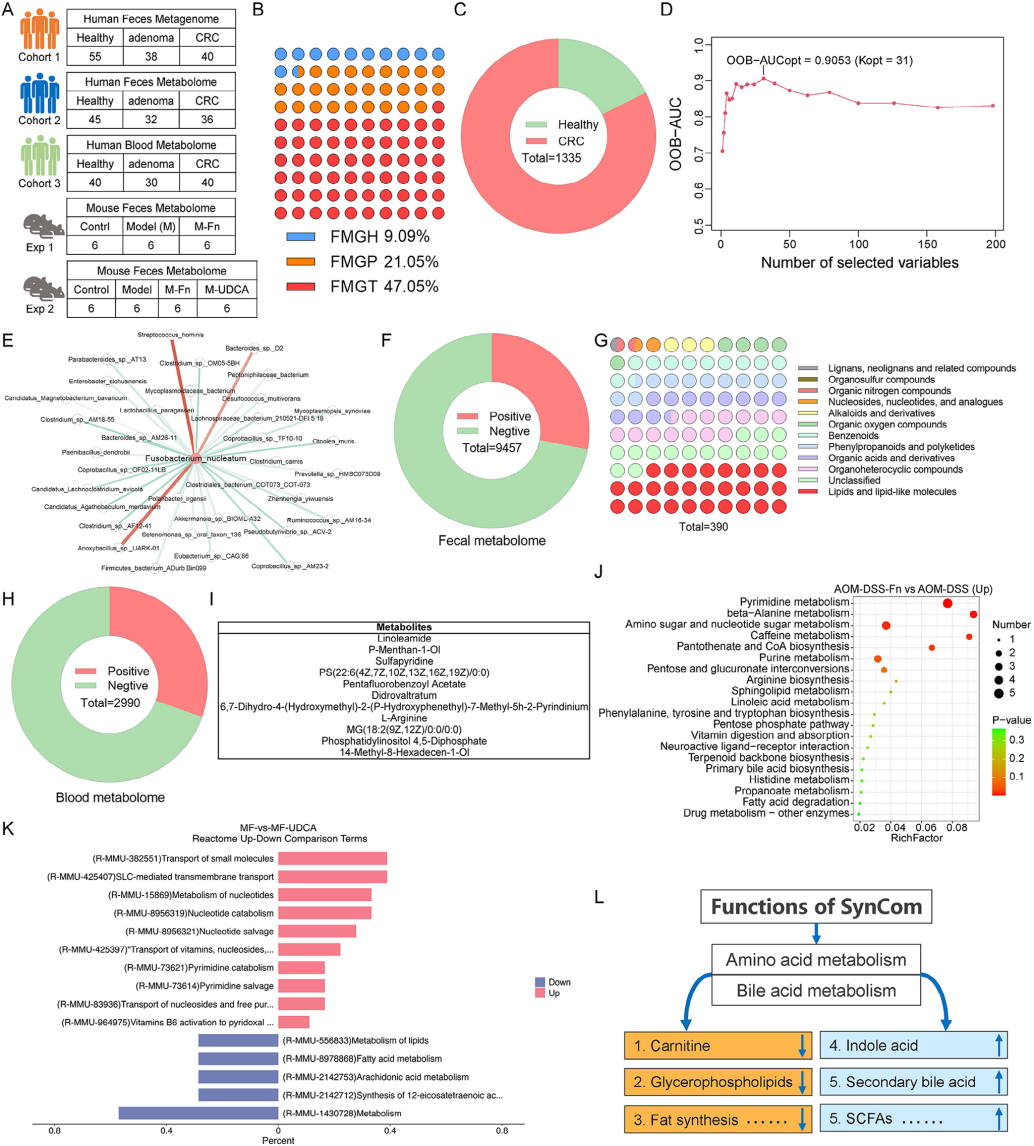
Analysis of *F. nucleatum*‐induced microbial and metabolic disorders. Three human cohorts and two mouse experiments were used for SynCom design A); percentages of high‐Fn samples in healthy, adenoma and CRC groups B); differential species distribution of human fecal metagenome (Kruskal‐Wallis test, *P* < 0.05) C); the random forest model with different microbial markers for Fn prediction D); Pearson correlation between Fn and 31 markers (*Bacteroides* sp. D2, *Streptococcus hominis* and *Anoxybacillus* sp. UARK‐01 were used as the control) E); the distribution of differential fecal metabolites (Kruskal‐Wallis test, *P* < 0.05) F); the classification of 390 metabolites that are positively correlated with Fn G); the distribution of differential blood metabolites (Kruskal‐Wallis test, *P* < 0.05) H); blood metabolites that are positively correlated with Fn (Pearson correlation, *P* < 0.05) I); upregulated KEGG pathways in the AOM‐DSS‐Fn group compared with the AOM‐DSS group (Fisher's exact test with Bonferroni correction) J); differential KEGG pathways between AOM‐DSS‐Fn and AOM‐DSS‐Fn‐UDCA groups (Fisher's exact test with Bonferroni correction) K); the potential functions of SynCom for *F. nucleatum* decolonization L).

To further discover commensal consortia to treat *F. nucleatum*‐infected CRC, we reanalyzed the metagenomics and metabolomics data in our previous study, including three human cohorts and two mouse experiments (**Figure** **2**A). According to the positive rate of Fn (45.38%) in CRC patients,^[^ ^31^ ^]^ we classified samples with absolute abundance greater than 20 as high‐Fn samples (accounting for 47.05% of the CRC sampes). The results showed that high‐Fn samples increased from the healthy (9.09%) to adenoma (21.05%) and CRC (47.05%) groups (Figure 2B). Next, the significant differential species between high‐Fn CRC and healthy samples (set 1), and between low‐Fn CRC and healthy samples (set 2) were identified (*P* < 0.05). Overall, 1335 species were unique to set 1, among which 235 species were enriched in healthy samples and 1100 species were enriched in CRC samples (Figure 2C). Next, Pearson correlation was determined between these 235 species and Fn, and 220 species were found to be negatively correlated with Fn. After removing viruses and unclassified species, 198 bacteria remained and were used for random forest model construction. The optimal panel to predict Fn comprised 31 species with an AUC of 0.9053 (Figure 2D,E). When the samples were divided into the training and validation sets (7:3), the new random forest model based on these 31 species achieved an accuracy of 0.8542 (95% CI: 0.7224‐0.9393, sensitivity: 0.8667, specificity: 0.8485).

We further utilized fecal metabolomics data and selected samples that matched the metagenome (the fecal sample used for metabolomics and the fecal sample used for the metagenome were from the same patient). The results indicated that 9547 metabolites were significantly correlated with Fn (Figure 2F, P < 0.05). Of these, 390 metabolites were positively correlated with Fn, and most belonged to lipids and lipid‐like molecules (Figure 2G). The human fecal metabolomics data indicated that Fn mainly induced lipid metabolic dysfunction. Furthermore, there were 3374 metabolites negatively related with Fn as well as enriched in the healthy group, which included indole molecules, bile acid metabolites and unsaturated fatty acids (Figure S2A, Supporting Information). Moreover, the human blood metabolomics matching the metagenome were analyzed (the blood sample used for metabolomics and the fecal sample used for the metagenome were from the same patient). The results indicated that 11 metabolites were positively correlated with Fn and enriched in high‐Fn CRC samples (*P* < 0.05), mainly including lipids and lipid‐like molecules (Figure 2H,I). Metabolites negatively correlated with Fn and enriched in the healthy group mainly included unsaturated fatty acid (Figure S2B, Supporting Information).

Next, the metabolome data of two AOM‐DSS‐induced mouse CRC model experiments were used for verification. In Exp 1, Fn oral administration to CRC mice caused upregulation of pyrimidine, purine and sphingolipid metabolism as well as primary bile acid biosynthesis (AOM‐DSS‐Fn vs. AOM‐DSS) (Figure 2J), which was also verified in Exp 2 (Figure S2C,D, Supporting Information). Lipid metabolism is acknowledged to be mainly regulated by bile acids, which are closely correlated with gut microbiota, through conjugated bile acid hydrolysis and secondary bile acid conversion. In Exp 2, the mouse fecal metabolomics revealed that metabolism of lipids, fatty acid metabolism and arachidonic acid metabolism were altered after UDCA (ursodeoxycholic acid) treatment (Figure 2K).

Therefore, human fecal and blood metabolomics as well as mouse fecal metabolomics confirmed that Fn induced a lipid metabolism disorder, which could be alleviated by UDCA. Thus, to design a SynCom that can alleviate Fn‐infected CRC, the SynCom should have several core functions (Figure 2L). The commensal consortia predicted by random forest model can be further optimized based on the expected functions of the SynCom.

### 2.3 Design of a Minimal Microbial Community Through Metabolic Network Reconstruction

**Figure 3:**
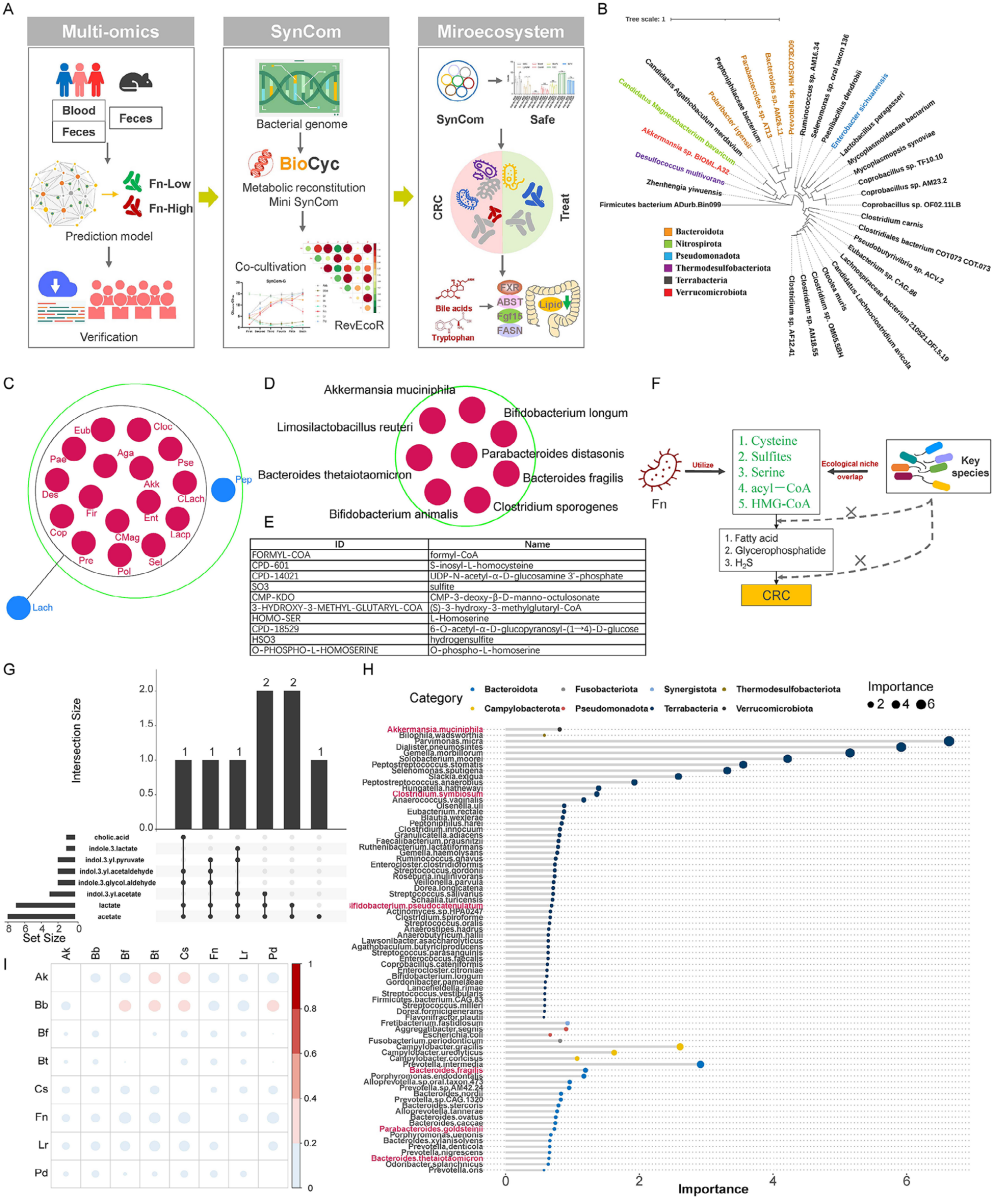
Design of the SynCom with specific functions. Framework of the SynCom design A); phylogenetic analysis of markers predicted by the random forest model B); the minimal microbial community analyzed by the Pathway Tools (dark pink for essential symbionts and blue for alternative symbionts) C); redundancy analysis of the designed SynCom D); the shared metabolites produced by SynCom in seed 1 and seed 2 E); ecological niche overlap between SynCom and Fn F); bile acid, tryptophan and SCFAs metabolites produced by the SynCom G); the random forest model of commensal consortia to predict *F. nucleatum* using fecal metagenomic sequencing data of curatedMetagenomicData H). complementation indces among SynCom members were calculated by the RevEcoR package using the KEGG Orthology information of the annotated genomic data from each species. The size and color of the point are proportional to the value of the index. (*Bifidobacterium animalis*, Bb; *Limosilactobacillus reuteri*, Lr; *Akkermansia muciniphila*, Ak; *Bacteroides thetaiotaomicron*, Bt; *Clostridium sporogenes*, Cs; *Parabacteroides distasonis*, Pd; *Bacteroides fragilis*, Bf) I).

To further reduce the functional redundancy of predicted commensal consortia, metabolic network reconstruction based on bacterial genomes was applied (**Figure** **3**A,B). Based on the pathway tool and Metage2Metabo workflow, eighteen species were selected as the minimal microbial community (Figure 3C). According to the expected functions of the SynCom (Figure 2L) and the culturability of these species, eight bacteria were selected with no redundancy in metabolism. These bacteria come from the gut of healthy individuals and their safety have been verified by previous studies (Figure 3D; Figure S3A, Supporting Information).

Ecological niche overlap is important for the SynCom to decolonize pathogenic microorganism, and we used two kinds of seeds to analyze the community metabolites. Seed 1 was reported in a previous study.^[^ ^32^ ^]^ To verify the conjugated bile acid hydrolysis ability of SynCom, seed 2 also contained taurocholic acid (TCA), glycocholic acid (GCA) and glycochenodeoxycholic acid (GCDCA). Free bile acid was successfully produced by SynCom (mainly by *B. animalis*) as expected. For the intersection of SynCom under the two seeds, the shared metabolites included sulfur‐containing substrates, glucose and amino acid metabolism intermediates. Fn is known to utilize L‐cysteine to produce hydrogen sulfide,^[^ ^33^ ^]^ and sulfite reductase allows it to generate sulfide from sulfite.^[^ ^34^ ^]^ Moreover, Fn can utilize glutamate, histidine, serine, and lysine as energy sources.^[^ ^35^ ^]^ Thus, the SynCom can compete with Fn to utilize nutritional substrates (Figure 3E,F). Additionally, most of the SynCom members could produce short‐chain fatty acids (SCFAs) and indole molecules from tryptophan metabolism (Figure 3G). These species were then verified using metagenomic data from curatedMetagenomicData. The random forest model was used to predict high‐Fn and low‐Fn samples, and the top 76 markers were listed according to their importance. In all, 62.5% (5/8) of the SynCom members were verified (Figure 3H). Finally, the competition and complementarity indices of SynCom+Fn were analyzed using RevEcoR based on the KEGG Orthology information of the annotated genomic data from each species. The result showed strong competition between Fn and Cs (Figure S3B, Supporting Information), and weak complementarity between Fn and other species (Figure 3I). Thus, the commensal consortium from the prediction model was optimized, and confirmed to demonstrate the expected functions, highlighting the potentiality of our design strategy.

### 2.4 Complex Competition and Complementarity Relationships among Syncom Members and *F. nucleatum*

**Figure 4:**
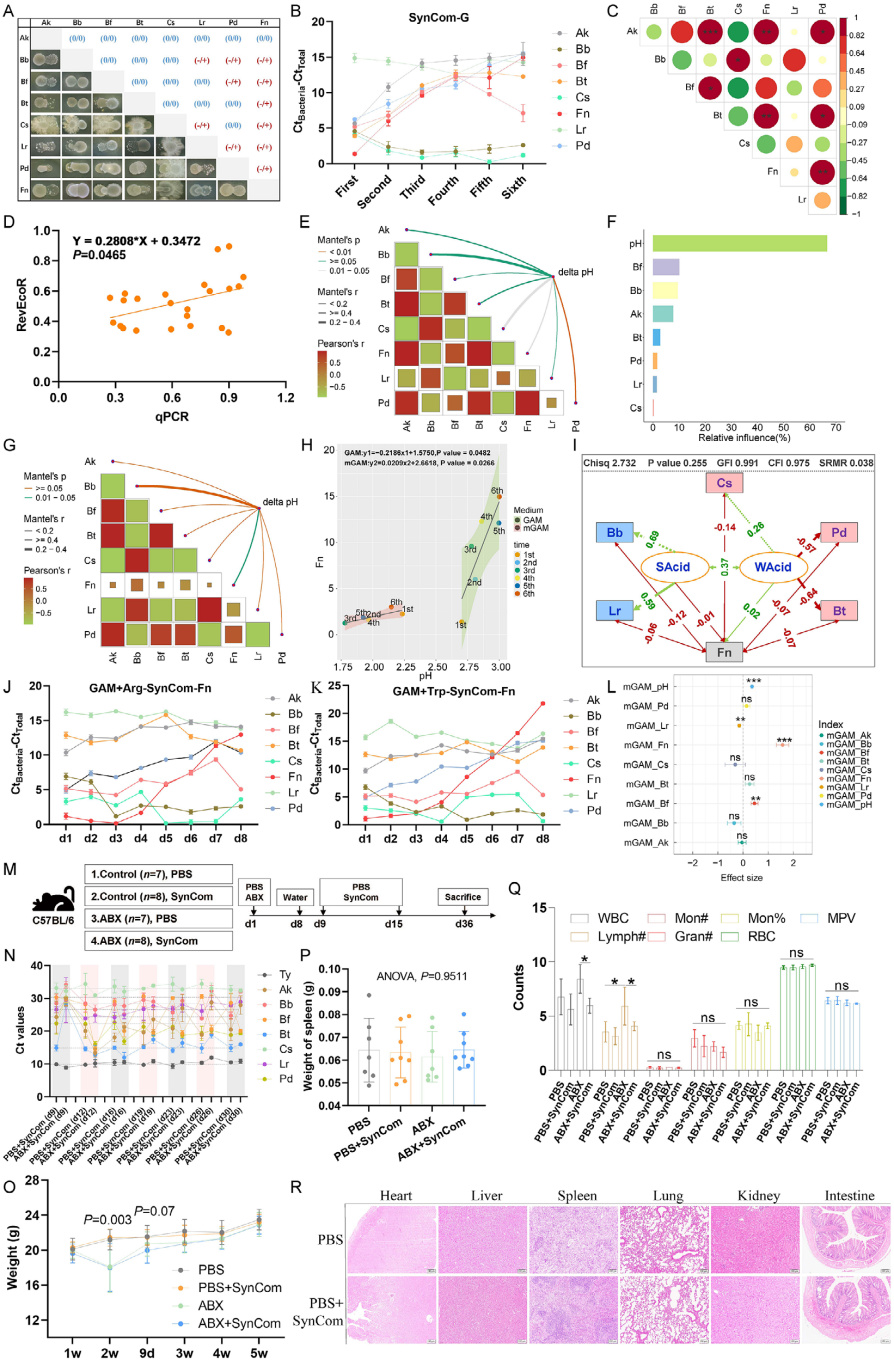
Complex competition and complementarity relationships among SynCom members and *F. nucleatum*. Co‐culture of the eight bacteria A); growth curves of SynCom in GAM medium. Data are mean ± SEM. *n* = 3 biologically independent experiments B); Pearson correlation analysis of SynCom in GAM medium. *n* = 6. *P < 0.05, **P < 0.01, **P < 0.001 by t‐test C); correlation between RevEcoR prediction and qPCR detection based on the correlation coefficient between each pair of the species D); correlation analysis between pH and SynCom members in GAM medium E); relative influence contribution of different indices on Fn abundance (caculated using gbmplus R package) F); correlation analysis between pH and SynCom members in mGAM medium G); linear fitting of pH and Fn abundance H); structural equation model of SynCom members and Fn using human fecal metagenomic sequencing data from cohort 1 (caculated using lmerTest and piecewiseSEM R packages) I); abundance changes of SynCom+Fn in GAM containing 1% arginine. Data are mean ± SEM. *n* = 3 biologically independent experiments J); abundance changes of SynCom+Fn in GAM containing 1% tryptophan. Data are mean ± SEM. *n* = 3 biologically independent experiments K); the response of Fn abundance to pH and to the abundance of other species. The random‐effect model is fitted based on the REML method. The vertical dashed line represents weighted effect size  =  0. Data are mean ± SE. **P < 0.01, **P < 0.001 by z test L); design of the normal mouse experiment M); SynCom colonization in vivo. Data are mean ± SEM N); body weight changes during the experiment. Data are mean ± SEM. P values by Student's t‐test represent the difference between PBS+SynCom and ABX+SynCom group O); spleen weights of different groups. Data are mean ± SEM P); blood routine analysis. Data are mean ± SEM. *P < 0.05 by Student's t‐test Q); Hematoxylin‐Eosin (HE) staining of the heart, liver, spleen, lung, kidney and intestine R).

The nine species were first cultured in vitro under anaerobic condition, and then *Bifidobacterium longum* (Bl) was removed because of its extremely low growth rate in GAM medium. Pairwise cultivation of the eight species was subsequently performed on GAM plates, and the result showed that the competition relationship only accounted for 23.8% (5/21) in the SynCom, but 85.7% (6/7) of them displayed strong competition relationship with Fn (**Figure** **4**A). When the seven species were inoculated in GAM at different proportions according to their growth rates, SynCom1, SynCom2, SynCom3 and SynCom4 were cultured for six generations and they showed similar growth patterns. All four SynComs reached a platform stage at the third generation (Figure S4A‐D, Supporting Information). When co‐cultured with SynCom in GAM, Fn gradually decreased and was negatively correlated with Bb and Cs (Figure 4B,C). Then the consistency between Pearson correlation and the competitive index calculated using RevEcoR was analyzed, and the liner fitting showed significant consistency (*P* = 0.0465, Figure 4D). To explore the potential mechanism, the pH of the six generations was monitored. The result showed that pH was negatively correlated with Fn abundance, and that pH changes made the highest contribution to the change in Fn abundance, accounting for 66.6% (Figure 4E,F). Additionally, we used mGAM to validate the influence of pH. After co‐culturing for eight generations of SynCom in mGAM, the negative correlation of pH and Fn was corroborated (Figure 4G,H). Next, we extracted bacterial abundance data from the human fecal metagenome sequencing data of cohort 1 (Figure 2A), and the linear mixed‐effect model and structural equation model showed that factors (bacteria) with strong acid‐production ability (Bb and Lr) had higher negative impacts on Fn (Figure 4I). However, Fn was found to reach the platform stage and maintain a high abundance in mGAM (Figure S4E, Supporting Information). Compared with GAM, arginine and tryptophan were added to mGAM. GAM‐SynCom and GAM‐SynCom+Fn showed similar pH curves, whereas pH was higher in mGAM‐SynCom (Figure S4F, Supporting Information). Thus, arginine and tryptophan were speculated to rescue Fn growth when cocultured with SynCom. Next, 1% arginine or 1% tryptophan were added to GAM. SynCom+Fn were cultured in Arg‐GAM and Trp‐GAM for eight generations. The results demonstrated that arginine and tryptophan weakened the acidity and slowed down the decline of Fn (Figure 4J,K; Figure S4G, Supporting Information). The effect size of the forest plot indicated that arginine and tryptophan had a significant negative impact on Lr and a positive impact on Fn (Figure 4L). Metabolomics data also showed that the metabolites in tryptophan and arginine metabolism pathways were increased in high‐Fn human fecal samples and in the feces of the AOM‐DSS‐Fn mice (Figure S4H–K, Supporting Information). Therefore, the in vitro co‐culture experiment displayed complex interactions among SynCom members and Fn, and confirmed the ability of the SynCom to resist *F. nucleatum*.

### 2.5 Colonization and Safety Evaluation of SynCom In Vivo

Considering the complex digestive tract conditions and differences between humans and mice, the SynCom was administered to mice by gavage. Furthermore, an antibiotic (ABX)‐treated group was included to analyze the influence of local organisms on the SynCom (Figure 4M). The colonization of the SynCom was monitored twice a week. Being different from in vitro culture, Bt, Pd, Bf, Ak, and Bf achieved colonization at a high abundance, whereas Cs was maintained at a low abundance. Moreover, ABX treatment did not enhance SynCom colonization (Figure 4N). In the process, the weight of mice in SynCom groups did not show any difference compared with that of the control group, whereas ABX treatment caused a significant weight decrease (Figure 4O). At the end of the experiment, the spleen weight in different groups was not significantly different (Figure 4P). Moreover, the blood test showed that ABX increased the white blood cell count (WBC), number of lymphocytes (lymph#), lymph%, and platelet count (PLT), whereas SynCom alleviated some of these inflammatory reactions (Figure 4Q; Figure S4L,M, Supporting Information). HE staining of the main organs also showed normal morphology (Figure 4R). Therefore, SynCom is safe in vivo and can achieve stable colonization.

### 2.6 SynCom Suppresses *F. nucleatum*‐infected CRC

**Figure 5:**
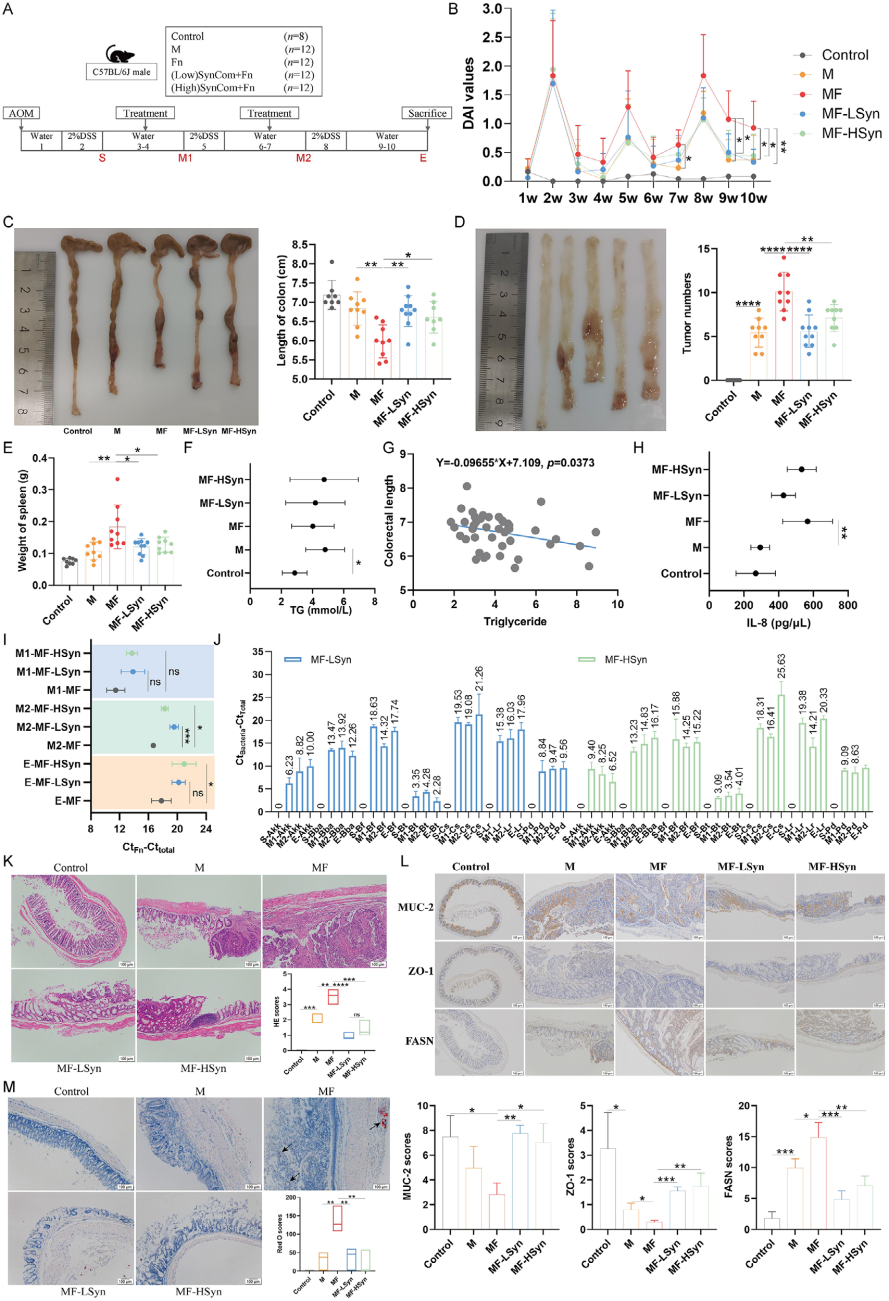
SynCom alleviates *F. nucleatum*‐infected CRC. Evaluation of SynCom in AOM‐DSS‐induced mouse CRC model A); DAI index changes during the experiment. Data are mean ± SEM. *P < 0.05, **P < 0.01 by Student's t‐test B); colon length of different groups. Data are mean ± SEM. *P < 0.05, **P < 0.01 by Student's t‐test C); tumor numbers of different groups. Data are mean ± SEM. **P < 0.01, ****P < 0.0001 by Student's t‐test D); spleen weight of different groups. Data are mean ± SEM. ****P < 0.0001 by Student's t‐test E); TG levels of the blood. Data are mean ± SEM. *P < 0.05 by Student's t‐test F); correlation between blood TG and colon length G); IL‐8 levels of different groups. Data are mean ± SEM. *P < 0.05 by Student's t‐test H); the abundance of Fn at the fifth (M1), eighth (M2) and tenth (E) weeks. Data are mean ± SEM. *P < 0.05, ****P < 0.0001 I); the colonization of SynCom at the S (third week), M1, M2 and E stages. Data are mean ± SEM J); HE staining analysis of the intestinal tissues. Data are mean ± SEM. *n* = 3. **P < 0.01, ***P < 0.001, ****P < 0.0001 by Student's t‐test K); IHC analysis of MUC‐2, ZO‐1 and FASN. Data are mean ± SEM. *n* = 3. *P < 0.05, **P < 0.01, ***P < 0.001 by Student's t‐test L); Oil red O staining analysis of the intestinal tissues. Data are mean ± SEM. *n* = 3. **P < 0.01 by Student's t‐test M).

Previous studies have demonstrated that *F. nucleatum* can promote CRC development, metastasis, and chemoresistance, and that eliminating Fn is beneficial for tumor inhibition.^[^ ^4^ ^]^ Thus, the treatment effect of SynCom was examined using the AOM‐DSS‐induced CRC mouse model, including Low‐SynCom and High‐SynCom groups (**Figure** **5**A). The disease activity index (DAI) showed that the gavage of Fn increased fecal occult blood, decreased weight and changed the fecal morphology, whereas the DAI values decreased significantly after SynCom treatment (Figure 5B). In addition, the AOM‐DSS treatment shortened the colon length and increased the tumor numbers, whereas SynCom mitigated this influence (Figure 5C,D). Furthermore, the weight of spleen obviously increased after Fn administration, but decreased in the SynCom groups (Figure 5E). The metabolomics analysis conducted above showed that Fn‐positive CRC patients and Fn‐infected mice showed enhanced lipid biosynthesis and energy metabolism, and the blood triglyceride (TG) test confirmed that TG in the MF group was higher than that in the M and control groups, but it declined in the SynCom groups though not significantly (Figure 5F). Additionally, TG was negatively correlated with colon length (Figure 5G). Previous studies confirmed that Fn promoted CRC proliferation through the IL‐8/TNF‐*α* pathway,^[^ ^36^ ^]^ and our study verified that blood IL‐8/TNF‐*α* levels were upregulated by Fn and downregulated by SynCom (Figure 5H; Figure S5, Supporting Information). Moreover, SynCom had negligible impacts on the levels of IgA, IgD, IgE, IgG and IgM in blood (Figure S5, Supporting Information). Next, the colonization of SynCom+Fn during the process was analyzed. The result showed reduced Fn abundance in the SynCom groups. In the M2 stage, Fn in MF was sixteen times higher than that in the Low‐SynCom group (Figure 5I). As for other species, they were below the detection limit at the S stage and colonized the gut at the M1 and M2 stages. Even after two weeks of oral administration, they still maintained colonization (Figure 5J). The colon HE staining results showed that Fn increased inflammatory cell infiltration, enhanced intestinal wall thickness and reduced goblet cells and crypt depths, whereas SynCom significantly alleviated these alterations (Figure 5K). More importantly, immunohistochemistry (IHC) and Oil red O staining showed that Fn treatment downregulated the expression of mucin protein (MUC‐2) and gut tight junction protein (ZO‐1), but increased the expression of fatty acid synthase (FASN) and lipid accumulation (Figure 5L,M). Liu et al. demonstrated that Fn promoted CRC cells to acquire stem cell‐like features through the FASN‐TLR4‐NF‐κB pathway.^[^ ^37^ ^]^ Our experiment in vivo confirmed that SynCom at least partially suppressed CRC development and reversed lipid accumulation by decolonizing *F. nucleatum*.

### 2.7 SynCom Alleviates *F. nucleatum*‐induced Carcinogenesis and Promotes Tryptophan and Bile Acid Metabolism

**Figure 6:**
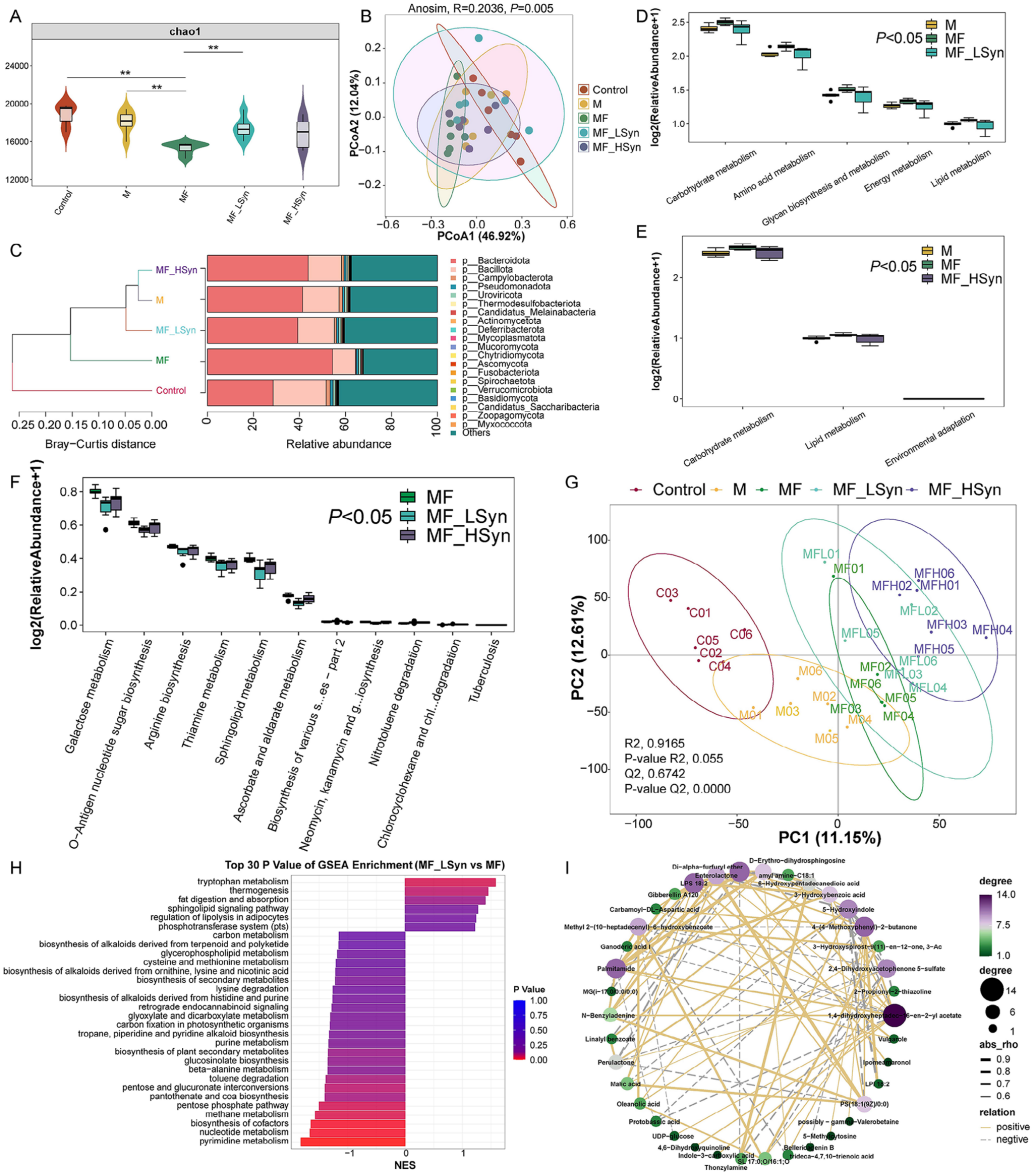
SynCom alleviates *F. nucleatum*‐induced microbial and metabolic disorders. Alpha diversity (Chao1 index) changes in different groups. Data are mean ± SEM. *n* = 6. **P < 0.01 by Student's t‐test A); PCoA of different groups. Data are mean ± SEM. *n* = 6. P value was calculated by PREANOVA test B); cluster analysis based on the bray‐curtis distance C); differential KEGG pathways of metagenome data among M, MF and MF_LSyn groups. Data are mean ± SEM. P values were calculated by Kruskal‐Wallis test D); differential KEGG pathways of metabolomics data among M, MF and MF_HSyn groups. Data are mean ± SEM. P values were calculated by Kruskal‐Wallis test E); differential species of metagenome data among MF, MF_LSyn and MF_HSyn groups. Data are mean ± SEM. P values were calculated by Kruskal‐Wallis test F); Partial Least Squares Discrimination Analysis (PLS‐DA) based on metabolomics G); GSEA enrichment analysis based on metabolomics between MF_LSyn and MF groups (NES, normalized enrichment score) H); the correlation network analysis of the differential metabolites between MF_LSyn and MF groups I).

To further explore the regulatory capability of SynCom on the gut microenvironment, mouse fecal metagenome sequencing and untargeted metabolomics were performed. First, the alpha diversity (Chao 1 index) was found to be decreased from the Control group to the M and MF groups, but was increased in the SynCom groups (**Figure** **6**A). Principal coordinates analysis (PCoA) showed that samples in the M and MF groups gradually moved away from the Control group, whereas samples in the SynCom groups were restored to the level before Fn infection (Figure 6B). Based on community composition at the phylum level, the MF_HSyn and MF_LSyn groups were clustered with the M group, being obviously different from those in the MF group (Figure 6C). At the genus level, *Duncaniella*, *Ligilactobacillus*, *Heminiphilus*, *Paramuribaculum*, *Roseburia*, *Oscillibacter*, and *Eubacterium* were more abundant in the Control, M, MF_LSyn, and MF_HSyn groups, yet *Paraprevotella*, *Bacteroides*, *Phocaeicola* and *Prevotella* were more abundant in the MF group (Figure S6A, Supporting Information). At the species level, strains of *Muribaculum* were increased after treatment (Figure S6B, Supporting Information). According to KEGG annotations of the metagenome data, cofactors and vitamins, lipid, energy, amino acid, carbohydrate, and glycan metabolism were upregulated in the MF group, indicating that Fn gavage promoted CRC development as well as related energy generation (Figure S6C,D, Supporting Information). However, SynCom treatment inhibited Fn‐induced lipid metabolism (Figure 6D,E). In particular, the metagenome results showed that arginine biosynthesis and sphingolipid metabolism was upregulated after Fn infection but downregulated in the SynCom groups, which met our design purpose (Figure 6F). As indicated by the procrustes analysis, both community composition and metabolic function of the microbiome were clearly different among the five groups (Figure S6E, Supporting Information), and fungal community alterations showed a distribution pattern similar to that of the bacterial community (Figure S6F, Supporting Information). In terms of fecal metabolomics changes, the PLSDA showed a similar trend with the PCoA of the metagenome (Figure 6G). Compared with the MF group, tryptophan metabolism and lipolysis regulation in adipocytes were upregulated in the MF_LSyn group, whereas nucleotide metabolism, pyrimidine metabolism, glycerophospholipid metabolism, and pyrimidine metabolism were downregulated (Figure 6H), and secondary bile acid biosynthesis, lipolysis regulation in adipocytes, and primary bile acid biosynthesis were enriched in the MF_HSyn group (Figure S6G, Supporting Information). Compared with MF_LSyn, MF_HSyn enhanced secondary bile acid biosynthesis, sulfur metabolism, and arginine and proline metabolism, which also met our design purpose (Figure S6H, Supporting Information). For instance, lipopolysaccharide 18:2, phosphatidylserine [18:1(9Z)/0:0], and palmitamide were downregulated by MF_LSyn, whereas 5‐hydroxyindole was upregulated (Figure S6I, Supporting Information). Similarly, gamma‐glutamylleucine and fatty acyls 18:1+30 were reduced after MF_HSyn treatment (Figure S6J, Supporting Information). According to the correlation network of differential metabolites between MF_LSyn and MF, 5‐hydroxyoxindole was negatively correlated with LPI 18:2, PS (18:1(9Z)/0:0) and 4‐(4‐methoxyphenyl)‐2‐butanone, whereas palmitamide was positively correlated with LPS 18:2 and D‐erythro‐dihydrosphingosine (Figure 6I).

**Figure 7:**
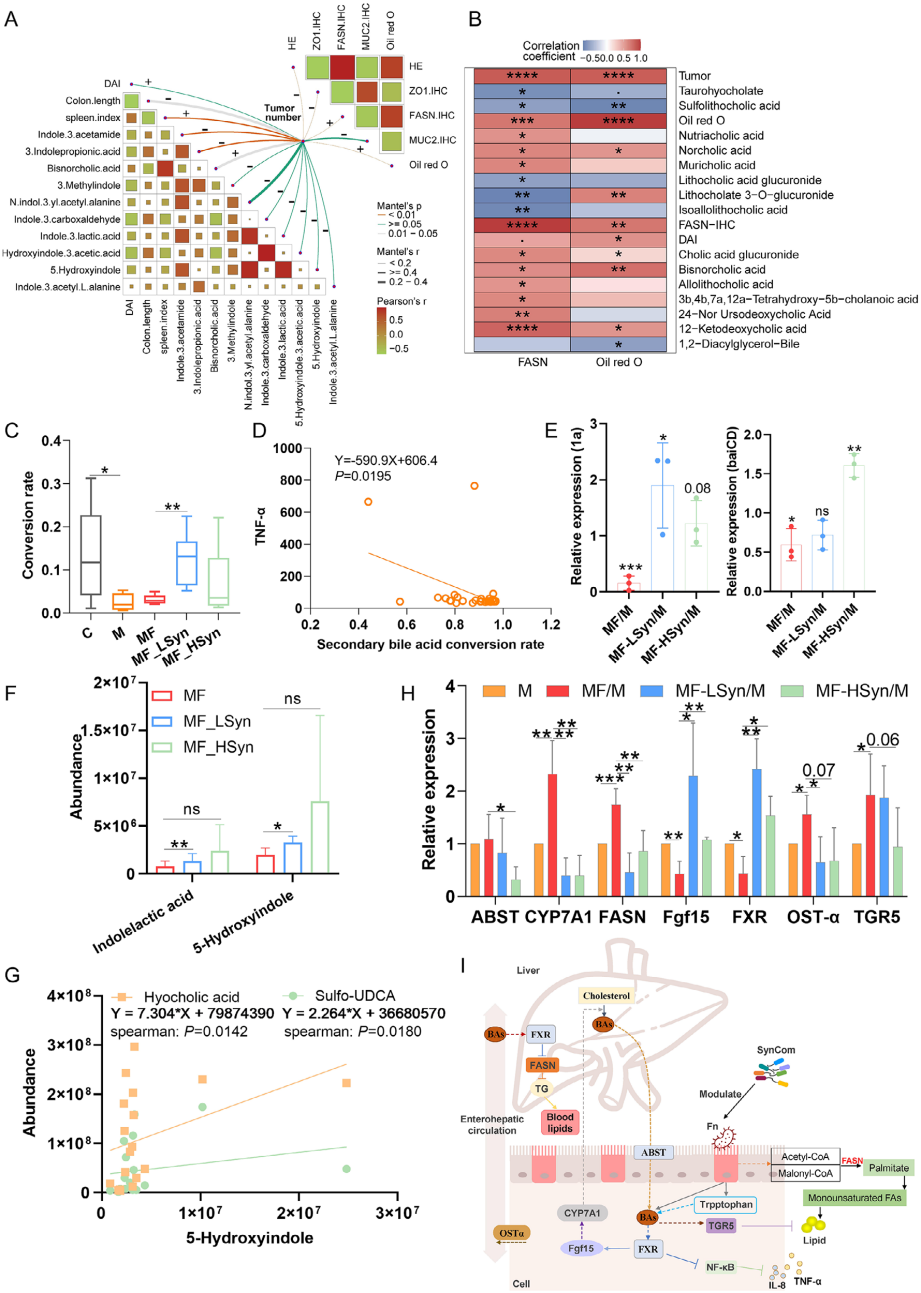
Correlation analysis among tryptophan metabolism, bile acid metabolism and lipid metabolism. Pearson correlation analysis among tumor numbers and tryptophan metabolites, bile acid metabolites and pathological indicators. (+, represent positive correlation; ‐, represent negative correlation) A); Pearson correlation analysis of FASN/Oil red O and bile acid metabolites. Data are mean ± SE. *P < 0.05, **P < 0.01, ***P < 0.001, ****P < 0.0001 by Student's t‐test B); bile acid conversion rate [lithocholic acid/(lithocholic acid+conjugated lithocholic acid)]. Data are mean ± SEM. *n* = 6. *P < 0.05, **P < 0.01 by Student's t‐test C); the correlation between secondary bile acid conversion rate and blood TNF‐*α* D); Bile acid metabolism related genes expression in intestinal tissues. Data are mean ± SEM. *n* = 3. *P < 0.05, **P < 0.01, ***P < 0.001 by Student's t‐test E); the abundance of indolelactic acid and 5‐hydroxyindole in MF, MF_Lsyn and MF_Hsyn groups. Data are mean ± SEM. *n* = 6. *P < 0.05, **P < 0.01 by Student's t‐test F); the correlation between 5‐hydroxyindole and hyocholic acid, and the correlation between 5‐hydroxyindole and sulfo‐UDCA G); the abundance of bile acid hydrolysis related bacterial genes in feces. Data are mean ± SEM. *n* = 6. *P < 0.05, **P < 0.01, ***P < 0.001 by Student's t‐test H); the regulation network of lipid metabolism by bile acid metabolism and tryptophan metabolism I).

Next, we analyzed the tryptophan metabolites and bile acid metabolites (they are related with lipid metabolism regulation by the gut microbiome). Interestingly, metabolites from tryptophan metabolism were negatively correlated with tumor number and DAI index, but were positively correlated with colon length. Bisnorcholic acid was positively correlated with tumor number and DAI index. Moreover, the tumor number was positively correlated with FASN expression and Oil red O result, but was negatively correlated with MUC‐2 and ZO‐1 expression (Figure 7A). We then extracted the bile acid metabolites at the idms1 level. The result showed that some free secondary bile acids and primary bile acids were positively correlated with FSAN and Oil red O data, whereas conjugated secondary bile acid showed a negative correlation with FASN and Oil red O data (Figure 7B). For example, the lithocholic acid (LCA) conversion rate [conjugated LCA/(free LCA+conjugated LCA)] was lower in the MF group, but increased after SynCom treatment (Figure 7C). Additionally, the secondary bile acid conversion rate was negatively correlated with blood TNF‐*α* levels (Figure 7D). Compared with that in the M group, qPCR detection showed that bacterial genes for secondary bile acid generation, such as 1a and baiCD, were decreased in the MF group, and increased in the SynCom groups (Figure 7E). Regarding tryptophan metabolites, indolelactic acid and 5‐hydroxyindole increased from MF to MF_LSyn and MF_HSyn groups, and 5‐hydroxyindole was positively related to hyocholic acid and sulfo‐UDCA (Figure 7G). Finally, genes related to bile acid transport and the bile acid‐lipid axis in intestinal tissues were analyzed using qPCR. The results showed that *ABST*, *CYP7A1*, *FASN*, *OST‐α* and *TGR5* were upregulated by Fn, whereas *Fgf15* and *FXR* were downregulated (Figure 7H). These influences were alleviated by SynCom, indicating that SynCom may decrease primary bile acid reabsorption (*OST‐α* and *ABST*), activate the FXR/Fgf15 pathway to promote bile acid synthesis and reduce blood TG. Furthermore, SynCom altered *FXR* and *TGR5* expression, which is known to be related to IL‐8, TNF‐*α* and lipid regulation (Figure 7I).

Overall, SynCom achieved colonization in the mouse CRC model, and mitigated microbiome dysbiosis, tryptophan and bile acid metabolic disorders, thus decolonizing *F. nucleatum*, reducing lipid accumulation and suppressing CRC development.

## 3 Discussion

In this study, *F. nucleatum* was proved to be increased in multiple fecal metagenomic sequencing cohorts, and it was also enriched in tumor tissues and induced broad alterations, such as gene expression, demethylation of oncogenes, lipid metabolism and so on. Using the machine learning model and metabolic network reconstruction, penitential commensal consortia were identified and optimized to obtain the minimal microbial community. Their interactions, ability to decolonize *F. nucleatum*, in vivo safety, and ability to regulate the microbiome and lipid metabolism were verified.

The human gut microbiota is composed of fungi, bacteria, viruses, and protozoa, where microbe‐microbe and microbe‐host interactions are essential for human health. The imbalance of these interactions, reflected by changes in composition and function, is associated with disease.^[^ ^38^ ^]^ Our metabolomics data revealed that the metabolic disorders induced by Fn involved several aspects. For example, glycerophospholipids, fat synthesis, and cholesterol synthesis increased. Indole organic acids were reduced. Secondary bile acid conversion and carnitine metabolites decreased. Phenylalanine, argisonyl‐glycyl‐aspartyl‐valine, and cysteine metabolism increased. The designed SynCom alleviated Fn‐infected CRC via several core functions, including hydrolysis and conversion of bile acid, tryptophan metabolism for indole compounds, and utilization of sulfur‐containing substrates. Furthermore, the co‐culture experiment also showed that Bb, Bf, Bt, Cs, Lr and Pd can inhibit Fn in the exploitation (+/–) manner. Therefore, alleviating the microbial and metabolic disorders via ecological control is a promising strategy.

In the co‐cultivation experiment, we found that the acidic environment and lower pH induced by SynCom is essential for Fn elimination. Fn is reported to be sensitive to acid. Its growth is inhibited at pH below 4.5 and have the maximum growth activity at pH 7.^[^ ^39^ ^]^ Human gut microbes can metabolize carbohydrates to produce SCFAs, whereas carbohydrates are also utilized by asaccharolytic species like Fn in which, glucose is used for the biosynthesis of intracellular macromolecules and not energy metabolism. Therefore, Fn utilizes nitrogenous substances for energy, is usually weakly fermentative, and tends to increase the local pH.^[^ ^40^ ^]^ Moreover, the enzyme activities of Fn, such as H_2_S‐producing enzymes, are higher in neutral condition.^[^ ^41^ ^]^ To survive hostile environmental conditions, Fn co‐adheres and forms a homogeneous biofilm at a growth pH of 8.2.^[^ ^42^ ^]^ Compared with growth at pH 7.4, Fn cultured at pH 8.2 produces more proteins associated with the metabolic enzymes and transport.^[^ ^43^ ^]^ Fn also upregulates fusobacterial oxygen‐induced sRNA,^[^ ^44^ ^]^ and FnFabM gene expression to resist acidic stress.^[^ ^45^ ^]^ Therefore, the acidic metabolites produced by SynCom play an important role against Fn.

Another interesting result is that arginine and tryptophan weaken the acidity and hinder the decline of Fn (Figure 4J,K; Figure S4G, Supporting Information). Arginine can be utilized by oral inhabitants as a substrate of the arginine deiminase system, eventually producing ATP and NH_3._^[^ ^46^ ^]^ Similarly, arginine can be degraded by the bifunctional ornithine decarboxylase (EC 4.1.1.17)/arginase (EC 3.5.3.1) enzyme (FN0501) in Fn, which yields ornithine and urea. Urea is degraded to ammonia and CO_2_.^[^ ^35^ ^]^ Tryptophan can be utilized by Fn (tnaA enzyme) to produce indole.^[^ ^47^, ^48^ ^]^ However, tryptophan can also be utilized by Bt, Bf and Cs to produce indole‐3‐acetic acid. Tryptophan can be utilized by Pd to produce indole‐3‐acrylic acid. Tryptophan can be utilized by *Bifidobacterium* and Lr to produce indole‐3‐lactic acid.^[^ ^49^ ^]^ Thus, Fn competes for tryptophan to produce indole instead of IAA, avoiding acid stress. Furthermore, the tryptophan degradation product indole can affect biofilm formation in various bacteria. Exogenous tryptophan and indole can increase Fn biofilm formation in a dose‐dependent manner,^[^ ^50^ ^]^ which enhances its ability to resist acid stress. The metabolomics data also confirmed that arginine metabolism was upregulated after Fn treatment. Arginine can be metabolized into NO and citrulline by nitric oxide synthase, into ornithine and urea by arginase, and into agmatine by arginine decarboxylase,^[^ ^51^ ^]^ which can be consumed by tumor cells. Moreover, Fn can produce arginine‐binding proteins to synthetize arginine‐inhibitable adhesins and increase co‐aggregation with other species to form a biofilm.^[^ ^52^ ^]^ Arginine resources are mainly derived from arginine‐enriched nutrition supplements from dietary intake, and via endogenous synthesis from citrulline and protein catabolism. Therefore, the influence of diet on SynCom deserves further exploration.

The most important metabolic disorder induced by Fn is correlated with lipid metabolism. Lipid metabolism disorder is a common feature of many cancers. Lipids can be funneled into diacylglycerides (DAGs) and triacylglycerides (TAGs), or converted into phosphoglycerides, such as phosphatidic acid (PA), phosphatidylethanolamine (PE) and phosphatidylserine (PS), thus providing energy for tumor cells.^[^ ^53^ ^]^ The metabolomics analysis in this study indicated that Fn infection increased the production of lipid metabolites, such as glycerophosphatide, and blood TG as well as FASN expression in the tumor. Therefore, targeting lipid metabolism may be beneficial for Fn‐positive CRC treatment. Interestingly, UDCA treatment to Fn‐infected mice reduced cholesterol and fatty acid, and SynCom increased the expression of the *baiCD* gene, which is essential for UDCA generation.^[^ ^54^ ^]^ Moreover, SynCom increased the production of SCFAs and the abundance of *Lactiplantibacillus*, which can modulate glucagon‐like peptide and bile salt deconjugation to produce a lipid‐lowering effect.^[^ ^55^ ^]^

Besides lipid and bile acid regulation, tryptophan metabolism enhancement is another important feature of SynCom. Tryptophan can be metabolized through serotonin (5‐hydroxytryptamine), kynurenine, and indole derivative pathways.^[^ ^56^ ^]^ In particular, microbiota‐derived indole derivatives were reported to promote chemopreventive effects and immune checkpoint inhibitor (ICI) treatment. Mackenzie et al. found that indole‐3‐aldehyde (I3A) derived from *L. reuteri* could induce aryl hydrocarbon receptor‐dependent CREB activity, and promote ICI response and survival in melanoma patients.^[^ ^57^ ^]^ Similarly, indole‐3‐lactic acid (ILA) from *L. reuteri* could suppress colorectal tumorigenesis by inhibiting T helper 17 cell differentiation to promote the atorvastatin treatment effect.^[^ ^58^ ^]^ The SynCom in this study increased indole‐3‐acetamide, 3‐indolepropionic acid, indole‐3‐lactic acid, and 5‐hydroxyindole‐3 acetic acid, and all of them showed a negative correlation with tumor number. Therefore, the application of SynCom in combination with other therapies may be an attractive direction.

Currently, only two products based on microbiome modulation for *C. difficile* infection (CDI) have been approved by FDA. The approval of REBYOTA and SER109 indicated that the direction of microbiome therapy is gradually transitioning from fresh feces to controllable microbiome construction.^[^ ^15^, ^59^ ^]^ In the clinicaltrials.gov website, most microbiome‐related clinical trials are focused on CDI treatment.^[^ ^60^ ^]^ Recently, microbiome therapy toward other pathogenic bacteria has been explored, such as utilization of SynCom for *Listeria monocytogenes*, *Enterococcus*, *K. pneumoniae* and *S. Typhimurium* elimination.^[^ ^16^, ^21^, ^61^ ^]^ In this study, we designed a tailored functional SynCom for Fn‐infected CRC treatment. However, the development of SynCom‐based therapies is in its infancy and several challenges need to be overcame. Most gut microbes have not been successfully cultured. The largest database of cultivated human gut bacteria only contains 3324 strains from eight phyla.^[^ ^62^ ^]^ Both the top‐down approach and and bottom‐up strategy are dependent on the selection of cultivated microbes, and cultureomics needs to be developed. The current metagenome sequencing is dependent on the sequence annotation, whereas current databases contain limited information about fungi, viruses and archaea, which are also reported to be important in CRC.^[^ ^63^, ^64^, ^65^ ^]^ Thus, the current databases need to be expanded. Another important issue is the analyses of multiomics data. Although massive amounts of sequencing data are available nowadays, paired and longitudinal study cohorts remain limited hindering comprehensive analysis. In terms of applying SynCom in the clinic, its stability during passage through the digestive tract needs to be improved by encapsulation in bioactive materials.^[^ ^66^ ^]^ Furthermore, microbes participate in the digestion of food and drug metabolism.^[^ ^67^, ^68^ ^]^ Therefore, their reactions to diet and medication should be assessed and monitored.

## 4 Experimental Section

### Metabolic Network Reconstruction and Minimal Microbial Community Design

The gbff genomic files of each bacterium were downloaded from NCBI and the seeds files was reported in previous studies.^[^ ^32^, ^69^ ^]^ All the work was performed following the m2m workflow. Simply, genomes and seeds were prepared as m2m inputs, and m2m recon ran metabolic network reconstruction for all annotated genomes using Pathway Tools.^[^ ^70^ ^]^

### Mouse CRC Model

C57BL/6 mice (6–8 weeks old) were purchased from Lanzhou Veterinary Research Institute and the experiment was approved by the Ethical Review Board of Lanzhou University. The CRC model was established using AOM (azoxymethane, Sigma, 10 mg kg^−1^) intraperitoneal injection (i.p.) and three rounds of 2% DSS (dextran sulfate sodium salt, Macklin, 36–40 kDa) drinking water. The SynCom was cultured in GAM medium. It was centrifuged at 4000 rpm for 10 min and washed with PBS. The bacterial precipitate was resuspended with PBS and adjusted to OD_600nm_ = 1.0. Similarly, Fn was cultured in GAM medium, centrifuged at 4000 rpm for 10 min, washed with PBS and adjusted to OD_600nm_ = 1.0. Next, Fn and SynCom were adjusted to the required concentration with PBS. Finally, for the SynCom treatment, SynCom was mixed with Fn before oral administration (200 µL for each mouse). The mice were divided into five groups: Control group, administered PBS by i.p., and PBS by gavage at the treatment stages (*n* = 8); Model (M) group, administered PBS by gavage at the treatment stages (*n* = 12); MF group, administered Fn (1 × 10^9^ CFU mL^−1^) by gavage at the treatment stages (*n* = 12); MF‐LSyn group, administered Fn (1 × 10^9^ CFU mL^−1^) and SynCom (1 × 10^10^ CFU mL^−1^) by gavage at the treatment stages (*n* = 12); MF‐HSyn group, administered Fn (1 × 10^9^ CFU mL^−1^) and SynCom (2 × 10^10^ CFU mL^−1^) by gavage at the treatment stages (*n* = 12).

### Shotgun Metagenome Sequencing of Fecal Samples

Fecal DNA was extracted using the Fecal Genome DNA Extraction Kit (AU46111‐96, BioTeke,China), and DNA libraries were constructed using the TruSeq Nano DNA Library Preparation Kit‐Set (#FC‐121‐4001, Illumina, USA). The libraries were sequenced on an Illumina NovaSeq 6000 platform with PE150 (LC‐Bio Technology Co., Ltd.). Reads containing adaptor contamination, low quality bases and undetermined bases were removed using fastp.^[^ ^71^ ^]^ Quality filtered reads were aligned to the mouse genome to filter out host contaminations using Bowtie 2.^[^ ^72^ ^]^ Next, the remaining reads were used for de novo assembly and annotations of microbial functions and taxonomy using MEGAHIT.^[^ ^73^ ^]^ Coding regions (CDS) of the assembled contigs were predicted using MetaGeneMark,^[^ ^74^ ^]^ and clustered using MMseq2 to obtain unigenes.^[^ ^75^ ^]^ Taxonomic assessment and functional annotations (KEGG, GO, eggNOG and CAZy) were performed using Diamond.^[^ ^76^ ^]^

### Untargeted Metabolomics Analysis of Fecal Samples

The fecal metabolites were extracted with 80% methanol and stored at −20 °C for 30 min. After centrifugation at 20 000 g for 15 min, the supernatants were subjected to vacuum drying. The samples were redissolved with 80% methanol and used for liquid chromatography‐tandem mass spectrometry (LC‐MS/MS) analysis. All chromatographic separations were performed using an UltiMate 3000 UPLC System, and ACQUITY UPLC T3 column (100 mm × 2.1 mm, 1.8 µm, Waters, Milford, USA) was used for the reversed phase separation. The column oven was maintained at 40 °C. The flow rate was 0.3 mL min^−1^ (Solvent A, 5 mM ammonium acetate and 5 mM acetic acid; solvent B, Acetonitrile).

MS data pretreatments including peak picking, peak grouping, retention time correction, second peak grouping, and annotation of isotopes and adducts was performed using XCMS software.^[^ ^77^ ^]^ Ions were identified by combining retention time (RT) and m/z data, and were then annotated using KEGG and HMDB databases to obtain idms1. Furthermore, they were validated using an in‐house fragment spectrum library of metabolites to obtain idms2.

### Statistical Analysis

All statistical analyses were performed using R software and GraphPad Prism. All data were mean ± SEM unless otherwise specified. The fold change of gene expression, microbial abundance and metabolite abundance were used after log2 transformation. Differences among three groups were analyzed using the Kruskal‐Wallis test or one‐way ANOVA test. Differences between two groups were analyzed using the Wilcoxon test or two‐tailed Students’ t tests. P values < 0.05 were considered statistically significant. All experiments were performed with at least three biological replicates as also described in each figure legend.

### Ethical Statement

The study protocol was approved by the Regional Ethical Review Board of Lanzhou University, the First Hospital of Lanzhou University, the Second Hospital of Lanzhou University and the Third People's Hospital of Gansu Province (21YF5FA112, 2021A‐152, 2022‐02‐28, 2022‐03‐01).

## Conflict of Interest

The authors declare no conflicts of interest.

## Author Contributions

P.C. and Z.Z. conceived the project and contributed to experimental designs. Z.Z., M.Y., B.Z., H.F., Y.M., Y.L., Y.L., Z.C., Y.Z., Z.S., and H.Z. performed experiments, generated figures and wrote the manuscript. P.C. and Z.Z. interpreted the results. P.C. supervised the project.

## Supporting information



Supporting Information

## Data Availability

The shotgun metagenome and whole genome sequencing data in this work have been deposited in the NCBI BioProject database (PRJNA1079906, PRJNA1086363, PRJNA1086371, PRJNA1182847 and PRJNA1214893). The metabolomics data in this work have been been deposited in the OMIX, China National Center for Bioinformation/Beijing Institute of Genomics, Chinese Academy of Sciences (OMIX008762, OMIX008772, OMIX008761, OMIX008758 and OMIX008965).
