# Supplementary material for: Tailoring a Functional Synthetic Microbial Community Alleviates Fusobacterium nucleatum‐infected Colorectal Cancer via Ecological Control
Source: Adv Sci (Weinh). 2025 May 28;12(31):e14232. doi: 10.1002/advs.202414232 (PMC12376641; doi:10.1002/advs.202414232)
Supplement: Supplementary file 1 — Supporting Information [file ADVS-12-e14232-s001.docx]

Supporting Information

Tailoring a Functional Synthetic Microbial Community Alleviates *Fusobacterium nucleatum*-infected Colorectal Cancer via Ecological Control

*Zhongkun Zhou, Mengyue Yang, Hong Fang, Baizhuo Zhang, Yunhao Ma, Yongyuan Li, Yingjie Liu, Zeying Cheng, Yuanchun Zhao, Zhenzhen Si, Hongmei Zhu, Peng Chen^*^*

Methods

**Fecal Metagenome Analysis of Public Data**

The metagenome sequencing data were downloaded from curatedMetagenomicData, which provides standardized, curated human microbiome data calculated by MetaPhlAn3 and HUMAnN3,^[^[^1^](#_ENREF_1)^]^ and curated sample metadata. The alpha diversity (Shannon index) was analyzed using the vegan R package, and the main phyla and genera were displayed using circlize and sourmashconsumr packages. The SparCC correlation was analyzed using SpiecEasi and pheatmap packages. Linear discriminant analysis Effect Size (LEfSe) analysis showing the differential species was performed on the Galaxy website. Related bacterial genes in KEGG pathways was also downloaded from curatedMetagenomicData for analysis.

**Tumor Microbiome Analysis Combined With Multiomics Data**

The tumor microbiome was obtained from The Cancer Microbiome Atlas (TCMA), which analyzed the prevalence of species across sample types from The Cancer Genome Atlas (TCGA). The whole-genome sequencing (WGS) data from samples in TCGA was screened for microbial content using the PathSeq pipeline.^[^[^2^](#_ENREF_2)^]^ Importantly, these data were filtered through strict decontamination process. Thus, the abundance of microbes combined with survival information were used to construct machine learning models. 16S rRNA sequences of the identified microbial markers were extracted from bacterial genome gbff files using Prokka software.^[^[^3^](#_ENREF_3)^]^ The sequence alignment and phylogenetic tree construction were performed using MEGA X,^[^[^4^](#_ENREF_4)^]^ and the phylogenetic tree was annotated using the ggtree package.^[^[^5^](#_ENREF_5)^]^ Furthermore, the DNA methylation (Methylation450k), gene expression RNAseq (IlluminaHiSeq), protein expression RPPA and somatic mutation data were downloaded from the UCSC Xena database. The matched samples between the UCSC Xena database and TCMA were used for correlation analysis. Those genes and proteins positively correlated with Fn were used for enrichment analysis using the clusterProfiler package.^[^[^6^](#_ENREF_6)^]^ The methylation site annotations were analyzed using the trackViewer package.

**Integrated Analysis of Metagenome and Metabolomics Data**

The metagenomics and metabolomics data of human feces and blood in our previous study were reanalyzed to explore potential commensal consortia against *F. nucleatum*, and metabolomics of mouse experiments were used for verification (PRJNA1079906, PRJNA1086363, PRJNA1086371, OMIX008770, OMIX008762, OMIX008772, OMIX008761 and OMIX008758). The differential species between different groups were identified by Kruskal Wallis test and Pearson correlation analysis. The potential commensal consortia were selected using random forest model, and further selected based on differential metabolic functions of bacterial genome.

**Mendelian Randomization Analysis**

The mendelian randomization analysis was performed using the TwoSampleMR R package, and the GWAS summary data was obtained from the IEU OpenGWAS project. The human blood metabolites were set as exposure variables^[^[^7^](#_ENREF_7)^]^. Meanwhile, Fn or CRC was set as the outcome variable. Single nucleotide polymorphism (SNPs) associated with exposure variables were extracted at the genome-wide significance level (p1 = 5×10^-8^). Next, r2 < 0.01 and kb = 10,000 were used to remove SNPs with chained disequilibrium. MR Egger regression, weighted median, inverse variance weighted, simple mode and weighted mode were used to test the importance of SNPs. Heterogeneity was evaluated by Q-test. Horizontal pleiotropy was assessed by se and p values. Statistical power and leave-one-out sensitivity analyses were performed for individual SNPs. Finally, the annotations of SNPs were obtained using the Ensembl GRCh37 website.

**Machine Learning Model Construction**

The differential species between different groups were firstly identified using Kruskal Wallis test, and these species were used for random forest model construction to predict high-Fn and low-Fn groups. The AUCRF package was used to identify the optimal combination of markers. The samples were then divided into a training set and a validation set (7:3). Next, a new random forest model was constructed and its performance was evaluated using randomForest and pROC packages.

For the survival model construction, the samples were first classified as high-Fn and low-Fn groups, and the samples were then divided into a training set and a validation set (8:2). Next, machine learning models, including random forest, ENet, Lasso, StepCox, CoxBoost, SuperPC, Grandient Boosting Machine, support vector machines, plsRcox, CoxBoost and their pairwise combinations, were evaluated.

**Metabolic Network Reconstruction and Prediction of The Minimal Microbial Community**

The gbff genomic files of each bacterium were downloaded from NCBI and the seeds files was reported in previous study.^[^[^8^](#_ENREF_8)^]^ All the work was performed following the m2m workflow. Simply, genomes and seeds were prepared as m2m inputs, and m2m recon ran metabolic network reconstruction for all the annotated genomes using Pathway Tools.^[^[^9^](#_ENREF_9)^]^ The standard output included reactions, compounds, genes, pathways, proteins and so on. Next, iscope module used seeds (SBML files) to produce reachable metabolites for each organism and all organisms as well as a matrix summarizing the producibility of molecules by these species. The cscope function produced molecules reachable by the community and detailed the roles of community members in the production of metabolites. The m2m addedvalue function generated added value of combining metabolisms in the microbiota. Most importantly, m2m mincom used files generated by m2m addedvalue to give the result of minimal community selection. The m2m analysis powergraph function created a graph to describe the minimal community (dark pink for essential symbionts and blue for alternative symbionts).

**Co-culture of Different Bacteria**

The *Fusobacterium nucleatum* (CGMCC 1.2526), *Bifidobacterium longum* (Bl, CGMCC 1.5082) and *Bifidobacterium animalis* (Bb, CGMCC 1.15623) were purchased from the China General Microbiological Culture Collection Center (CGMCC). *Limosilactobacillus reuteri* (Lr, GDMCC 1.614), *Akkermansia muciniphila* (Ak, GDMCC 1.1346), *Bacteroides thetaiotaomicron* (Bt, GDMCC 1.1104), *Clostridium sporogenes* (Cs, GDMCC 1.1481), *Parabacteroides distasonis* (Pd, GDMCC 1.1564) and *Bacteroides fragilis* (Bf, GDMCC 1.385) were purchased from the Guangdong Microbial Culture Collection Center (GDMCC). They were cultured in Gifu Anaerobic Medium (GAM) under anaerobic condition at 37 ^o^C. Bacteria in logarithmic growth phase were centrifuged for 10 min at 4000 rpm. The bacterial precipitate was resuspended in GAM and was adjusted to the same concentration (10^9^ CFU/mL). 10 μL of each bacterium was added to the GAM plate, being adjacent to another bacterium. The plates were cultured for 96 h at 37 ^o^C.

**Cultivation of Synthetic Microbial Communities**

The bacteria were cultured in GAM until logarithmic growth period, and then each bacterium was centrifuged at 4000 rpm for 10 min, and the precipitate was resuspended in medium. Next, they were adjusted to OD_600nm_=1.0 as seeds for further use. Bl was excluded because of its extremely slow growth rate in GAM. The SynComs were constructed at different inoculation proportions according to their growth rates (Lr+Bb+Bf+Cs+Ak+Bt+Pd+GAM: SynCom1, 0.05+0.05+0.05+0.05+0.05+0.05+0.05+0.65 mL; SynCom2, 0.10+0.10+0.10+0.10+0.10+0.10+0.10+0.30 mL; SynCom3, 0.05+0.05+0.05+0.05+0.05+0.10+0.10+0.65 mL; SynCom4, 0.05+0.05+0.05+0.05+0.05+0.10+0.20+0.45 mL). 1 mL of each SynCom was inoculated to 40 mL medium and it was marked as the first generation. 24 h later, each SynCom was centrifuged at 4000 rpm for 10 min. The precipitate was resuspended in medium and the supernatant was collected for pH detection. Next, they were adjusted to OD_600nm_=1.0, and 0.4 mL of the first generation was inoculated to 40 mL fresh medium and it was marked as the second generation. Furthermore, 1 mL of the bacterial fluid (OD_600nm_=1.0) was washed with PBS and used for genomic extraction as well as qPCR detection immediately. Each SynCom was continuously cultured for six to eight generations.

**Competition and Complementarity Indices Calculation**

The annotated genomic data of each species were downloaded from the JGI IMG Integrated Microbial Genomes & Microbiomes database (*Fusobacterium nucleatum*, Gc00085; *Limosilactobacillus reuteri*, Gs0016615; *Bifidobacterium animalis*, Gs0002217; *Akkermansia muciniphila*, Gs0000899; *Bacteroides thetaiotaomicron*, Gs0151297; *Clostridium sporogenes*, Gs0016050; *Parabacteroides distasonis*, Gs0019360; *Bacteroides fragilis*, Gs0002059). The KEGG Orthology (KO) profile of each bacterium was extracted, and the competition index and complementarity index were calculated using the RevEcoR R package.^[^[^10^](#_ENREF_10)^]^

The co-culture of SynCom and Fn was started with the inoculation proportion of SynCom2. The abundance of each bacterium in six generations was used for Pearson correlation calculation, and the correlation between competition index and Pearson's correlation was analyzed.

**Linear Mixed Effects Model and Structural Equation Model** **Construction**

The abundance data of Fn, Ak, Bb, Bf, Bt, Cs, Lr and Pd were extracted from the fecal metagenomic sequencing data (cohort 1). The linear mixed effects model was constructed using the lme4 package. Fn was set as the dependent variable and other bacteria were set as independent variables, with sex as the random effect variable. The formula was as follows: Fn ~ Ak+Bb+Bf+Bt+Cs+Lr+Pd+(1|sex), and the significance of each variable was calculated using ANOVA analysis.

The structural equation model was constructed using piecewiseSEM and lmerTest packages. Bb and Lr were classified as species with strong acid-production ability (SAcid), whereas Cs, Bt and Pd were classified as species with weak acid-production ability (WAcid). The significance of each variable was calculated using ANOVA test, and model indices (chisq, df, pvalue, gfi, cfi, rmr, srmr and rmsea) were calculated using the fitMeasures function.

**Safety Assessment *in vivo***

C57BL/6 mice (6-8 weeks old) were purchased from Lanzhou Veterinary Research Institute and the experiment was approved by the Ethical Review Board of Lanzhou University. They were divided into four groups: Control group was administered with phosphate buffer saline (PBS) by gavage (*n* = 7); Control-SynCom group was administered with PBS and SynCom (1×10^10^ CFU/mL) (*n* = 8); ABX group was treated with ABX-containing drinking water (0.5 mg/mL vancomycin, 1 mg/mL neomycin, 1 mg/mL metronidazole, and 1 mg/mL ampicillin) and was administered with PBS (*n* = 7); ABX-SynCom group was treated with ABX-containing drinking water and was administered with SynCom (1×10^10^ CFU/mL) (*n* = 8).

**Blood Routine Test**

The blood were collected using blood collection tubes containing EDTA, and the samples were detected using mindray (BC-2800Vet), including white blood cell count (WBC), number of lymphocytes (Lymph#), number of monocytes (Mon#), number of neutrophils (Gran#), percentage of lymphocytes (Lymph%), percentage of monocyte (Mon%), percentage of neutrophil (Gran%), red blood cell count (RBC), hemoglobin (HGB), hematocrit (HCT), mean corpuscular volume (MCV), mean corpuscular hemoglobin content (MCH), mean corpuscular hemoglobin concentration (MCHC), variation coefficient of red blood cell distribution width (RDW), platelet count (PLT), mean platelet volume (MPV), platelet distribution width (PDW), and plateletcrit (PCT).

**Hematoxylin-Eosin Staining of Tissues**

Paraffin embedded tissue slices were dewaxed and dehydrated using xylene and ethanol, respectively. The cell nucleus was stained using hematoxylin, and cytoplasm was stained using eosin. Finally, slices were dehydrated with ethanol and xylene again. The HE scores were calculated according to the following rules: infiltration of inflammatory cells (0-4), mucosal thickening (0-4), reduction of goblet cells (0-4), disappearance of crypts (0, 3, 4), structural damage (0, 3, 4).

**Disease Activity Index Evaluation**

The disease activity index evaluation was performed every week. The scoring rules were as follows: weight loss (0, 0%; 1, 1-5%; 2, 5-10%; 3, 10-20%; 4, >20%), feces (0, normal; 1, soft; 2, mucus-like; 3, liquid), fecal occult blood test (FOBT) (0, negative; 1, light purple; 2, purple; 3, deep purple; 4, bloody stool). Finally, the average score of three indices was used as the DAI values.

**Enzyme-linked Immunosorbent Assay**

The blood were collected, stored at room temperature for 30 min, centrifuged at 3000 rpm for 15 min, and stored at -80 ^o^C. The IL-8, TNF-α, IgA, IgD, IgE, IgG and IgM levels were detected using the Mouse IL-8 ELISA kit (SEKM-0046, Beijing Solarbio Science & Technology Co., Ltd.), TNF-α ELISA kit (SEKM-0034, Beijing Solarbio Science & Technology Co., Ltd.), Mouse Immunoglobulin A (IgA) ELISA Kit (ml037606, Shanghai Enzyme-linked Biotechnology Co., Ltd), Mouse Immunoglobulin D (IgD) ELISA Kit (ml037604, Shanghai Enzyme-linked Biotechnology Co., Ltd), Mouse Immunoglobulin E (IgE) ELISA Kit (ml037602, Shanghai Enzyme-linked Biotechnology Co., Ltd), Mouse Immunoglobulin G (IgG) ELISA Kit (ml057874, Shanghai Enzyme-linked Biotechnology Co., Ltd), and Mouse Immunoglobulin M (IgM) ELISA Kit (ml063597, Shanghai Enzyme-linked Biotechnology Co., Ltd) according to the manufacturers’ instructions.

**Quantitative Real-Time PCR**

The bacterial genome was extracted using the TIANamp Bacteria DNA Kit (DP302, TIANGEN BIOTECH CO.,LTD.). The tissue RNA was extracted using the Tissue Genomic DNA Extraction Kit (G3633, Servicebio) and reverse transcribed into cDNA with SweScript RT II First Strand cDNA Synthesis Kit (G3333, Servicebio) following the manufacturer’s instructions.

The qPCR reaction system contained 5 μL 2×SYBR Green qPCR Master Mix, 1 μL DNA or cDNA, 0.3 μL forward primer, 0.3 μL reverse primer, and 3.4 μL nuclease-free water. The reaction procedure was set as follows: 50 ^o^C for 2 min, 95 ^o^C for 30 s, 40 cycles of 95 ^o^C for 15 s and 60 ^o^C for 1 min. The primers were listed in Table S1, and the results were analyzed with the QuantStudio Design & Analysis Software.

**Immunohistochemistry Analysis**

Paraffin embedded tissue slices were dewaxed and dehydrated using xylene and ethanol, respectively. Antigen retrieval was performed using 0.01 mol/L sodium citrate, and endogenous peroxidase was blocked with hydrogen peroxide. The slices were then blocked using bovine serum albumin, and were incubated with antibodies (Anti-MUC2 Mouse mAb, 1:500, Servicebio; Anti-ZO1 tight junction protein Mouse mAb, 1:200, Servicebio; Anti -Fatty Acid Synthase Mouse mAb, 1:1000, Servicebi) and HRP-labeled secondary antibody. Next, slices were stained with diaminobenzidine, and cell nucleus was stained using hematoxylin. Finally, slices were dehydrated with xylene and ethanol.

**Oil Red O Staining Analysis**

The intestinal tissues were embedded with optimal cutting temperature compound and frozen with liquid nitrogen. Frozen slices were then washed with 60% isopropanol and stained with Oil red O. Next, they were washed with 60% isopropanol, and cell nucleus were stained using hematoxylin. The images were analyzed using the fluorescence microscope (Olympus, BX53+DP74).

**Blood Triglyceride Analysis**

The blood triglyceride was detected using the Triglyceride assay kit (A110-1-1, Nanjing Jiancheng Bioengineering Institute) according to the manufacturer’s instructions.

**Untargeted Metabolomics Analysis of Fecal Samples**

The fecal metabolites were extracted with 80% methanol and stored at -20 ^o^C for 30 min. After centrifugation at 20,000 g for 15 min, the supernatants were subjected to vacuum drying. The samples were redissolved with 80% methanol and used for LC-MS analysis. All chromatographic separations were performed using an UltiMate 3000 UPLC System, and ACQUITY UPLC T3 column (100 mm × 2.1 mm, 1.8 µm, Waters, Milford, USA) was used for the reversed phase separation. The column oven was maintained at 40 ^o^C. The flow rate was 0.3 mL/min (Solvent A, 5 mM ammonium acetate and 5 mM acetic acid; solvent B, acetonitrile).

High-resolution tandem mass spectrometer TripleTOF 6600 (SCIEX, Framingham, MA, USA) was used to detect the metabolites. The ion spray voltage floating was set at 5000 V and 4500 V for positive ion mode and negative ion mode, respectively. The TOF mass ranges from 60 to 1200 Da, and the survey scans were acquired in 150 ms. Dynamic exclusion was set as 4 s. A quality control sample (pool of all the samples) was acquired after every ten samples.

MS data pretreatments, including peak picking, peak grouping, retention time correction, second peak grouping, and annotation of isotopes and adducts, was performed using the XCMS software.^[^[^11^](#_ENREF_11)^]^ Ions were identified by the combining retention time (RT) and m/z data, and they were annotated using KEGG and HMDB databases to obtain idms1. Furthermore, they were validated using an in-house fragment spectrum library of metabolites to obtain idms2.

Pearson correlation analysis was performed using the cor package. Significant different metabolites were identified according to three conditions (P value < 0.05, difference multiple > 1.2 obtained by t test, and VIP calculated by PLSDA analysis). GSEA and MSigDB were used for gene set enrichment analysis. |NES| > 1, NOM p-value < 0.05 and FDR q-value < 0.25 were considered to be significantly different between two groups.

**Table S1. Primers used in the qPCR experiment.**

| **Name** | **Sequences** | **References** |
| --- | --- | --- |
| Lr-F | 5’-GGAGAAGAACGTGCGTGAGA-3’ | This study |
| Lr-R | 5’-AGCCGAAGGCTTTCACATCA-3’ | This study |
| Bt-F | 5’-GAGGAAGGTCCCCCACATTG-3’ | This study |
| Bt-R | 5’-ACCCATAGGGCAGTCATCCT-3’ | This study |
| Ak-F | 5’-CAGCACGTGAAGGTGGGGAC-3’ | ^[^[^12^](#_ENREF_12)^]^ |
| Ak-R | 5’-CCTTGCGGTTGGCTTCAGAT-3’ | ^[^[^12^](#_ENREF_12)^]^ |
| Cs-F | 5’-TTGGCTCTGCACCGGGAATC-3’ | ^[^[^13^](#_ENREF_13)^]^ |
| Cs-R | 5’-CTGCAAACGCCGTCCCTCTT-3’ | ^[^[^13^](#_ENREF_13)^]^ |
| Pd-F | 5’-CGGTTTCGTGCGGGTGATGA-3’ | ^[^[^13^](#_ENREF_13)^]^ |
| Pd-R | 5’-CGGAAGGTGGCGTTCGTGTT-3’ | ^[^[^13^](#_ENREF_13)^]^ |
| Bf-F | 5’-CACTTGACTGTTGTAGATAAAGC-3’ | ^[^[^14^](#_ENREF_14)^]^ |
| Bf-R | 5’-CATCTTCATTGCAGCATTATCC-3’ | ^[^[^14^](#_ENREF_14)^]^ |
| Bb-F | 5’-CACCAATGCGGAAGACCAG-3’ | ^[^[^15^](#_ENREF_15)^]^ |
| Bb-R | 5’-GTTGTTGAGAATCAGCGTGG-3’ | ^[^[^15^](#_ENREF_15)^]^ |
| Bl-F | 5’-CGGCGTYGTGACCGTTGAAGAC-3’ | ^[^[^15^](#_ENREF_15)^]^ |
| Bl-R | 5’-TGYTTCGCCRTCGACGTCCTCA-3’ | ^[^[^15^](#_ENREF_15)^]^ |
| 63-F | 5’-GCAGGCCTAACACATGCAAGTC-3’ | ^[^[^16^](#_ENREF_16)^]^ |
| 335-R | 5’-CTGCTGCCTCCCGTAGGAGT-3’ | ^[^[^16^](#_ENREF_16)^]^ |
| Fn-F | 5’-CAACCATTACTTTAACTCTACCATGTTCA-3’ | ^[^[^16^](#_ENREF_16)^]^ |
| Fn-R | 5’-GTTGACTTTACAGAAGGAGATTATGTAAAAATC-3’ | ^[^[^16^](#_ENREF_16)^]^ |
| GAPDH-F | 5′-GGAGCGAGATCCCTCCAAAAT-3′ | ^[^[^17^](#_ENREF_17)^]^ |
| GAPDH-R | 5′-GGCTGTTGTCATACTTCTCTCATGG-3′ | ^[^[^17^](#_ENREF_17)^]^ |
| baiCD-F | 5’-GGWTTCAGCCCRCAGATGTTCTTTG-3’ | ^[^[^18^](#_ENREF_18)^]^ |
| baiCD-R | 5’-GAATTCCGGGTTCATGAACATTCTKCKAAG-3’ | ^[^[^18^](#_ENREF_18)^]^ |
| 1a-F | 5’-CACATATTGTGGCACGAACAATHGARTGGGG-3’ | ^[^[^19^](#_ENREF_19)^]^ |
| 1a-R | 5’-CTGTGCCCGGATACAGATTAACRTARTTRTT-3’ | ^[^[^19^](#_ENREF_19)^]^ |
| 1b-F | 5’-CGGCGTTCCGCATTTYTAYGARAA-3’ | ^[^[^19^](#_ENREF_19)^]^ |
| 1b-R | 5’-GTTCAATGCCAATCGGAATATCRAARTTRTT-3’ | ^[^[^19^](#_ENREF_19)^]^ |
| 3c-F | 5’-TTTTGGCCGAACACTGGAYTAYGARTT-3’ | ^[^[^19^](#_ENREF_19)^]^ |
| 3c-R | 5’-TCAACGGAGCCCAGAATATGRAARAAYTG-3’ | ^[^[^19^](#_ENREF_19)^]^ |
| β-actin-F | 5’-ACCTGACAGACTACCTCATGAAGA-3’ | ^[^[^20^](#_ENREF_20)^]^ |
| β-actin-R | 5’-TCATGGATGCCACAGGATTCCATA-3’ | ^[^[^20^](#_ENREF_20)^]^ |
| Fxr-F | 5’-TGGGCTCCGAATCCTCTTAGA-3’ | ^[^[^20^](#_ENREF_20)^]^ |
| Fxr-R | 5’-TGGTCCTCAAATAAGATCCTTGG-3’ | ^[^[^20^](#_ENREF_20)^]^ |
| Fgf15-F | 5’-GCCATCAAGGACGTCAGCA-3’ | ^[^[^20^](#_ENREF_20)^]^ |
| Fgf15-R | 5’-CTTCCTCCGAGTAGCGAATCAG-3’ | ^[^[^20^](#_ENREF_20)^]^ |
| TGR5-F | 5’-TCCTGTCAGTCTTGGCCTATGA-3’ | ^[^[^20^](#_ENREF_20)^]^ |
| TGR5-R | 5’-GGTGCTGCCCAATGAGATG-3’ | ^[^[^20^](#_ENREF_20)^]^ |
| CYP7A1-F | 5’-AGCAACTAAACAACCTGCCAGTACTA-3’ | ^[^[^20^](#_ENREF_20)^]^ |
| CYP7A1-R | 5’-GTCCGGATATTCAAGGATGCA-3’ | ^[^[^20^](#_ENREF_20)^]^ |
| ASBT-F | 5’-GTGGGCTTCCTCTGTCAGTT-3’ | ^[^[^20^](#_ENREF_20)^]^ |
| ASBT-R | 5’-GCATCATTCCAAGGGCAAGC-3’ | ^[^[^20^](#_ENREF_20)^]^ |
| OSTa-F | 5’-CTGAAGGACACCCCGATGAG-3’ | ^[^[^20^](#_ENREF_20)^]^ |
| OSTa-R | 5’-CCTGGGTCATAGATGCCGTC-3’ | ^[^[^20^](#_ENREF_20)^]^ |
| FASN-F | 5’-GAGTTCTCAGGCCGGGATAGGT-3’ | ^[^[^20^](#_ENREF_20)^]^ |
| FASN-R | 5’-TGGTATAGACGACGGGCACAGA-3’ | ^[^[^20^](#_ENREF_20)^]^ |

**Supplementary figure captions**

**Figure S1.** *F. nucleatum* is enriched in CRC patients and correlated with lipid metabolism disorder. The upregulated genes of *F. nucleatum* in CRC patients based on the KEGG annotations of metagenome from curatedMetagenomicData (**P* < 0.05, ***P* < 0.01, ****P* < 0.001 by Student’s t-test) (A); Non-metric multidimensional scaling (NMDS) analysis of healthy individuals and CRC patients (k = 3 for NMDS and PERMANOVA test was based on the Euclidean distance) (B); heat tree of the most abundant 40 genera (C); the abundance of species of *Fusobacterium*. Data are mean ± SEM. P values were calculated by Student’s t-test (D); the microbial markers for survival prediction of CRC patient (alive and deceased) (E); the survival curve of *B. fragilis*-high (abundance ≥ 55) and *B. fragili*s-negative (abundance = 0) groups. P values were calculated by Log-Rank test (F); machine learning models for survival time prediction based on tumor microbiome markers (training set : validation set = 8:2) (G); the enrichment analysis of proteins that are positively correlated with Fn (*P* < 0.05 by t-test of Pearson correlation) (H); MR effect size for Fn on CRC. Error bars represent 95% confidence intervals (CIs) (I).

**Figure S2.** Prediction model of commensal consortia for *F. nucleatum* decolonization. The enrichment analysis of human fecal metabolites that are negatively correlated with Fn and enriched in healthy individuals (*P* < 0.05 by t-test of Pearson correlation) (A); the enrichment analysis of human blood metabolites that are negatively correlated with Fn and enriched in healthy individuals (*P* < 0.05 by t-test of Pearson correlation) (B); differential KEGG pathways of metabolomics between AOM-DSS and AOM-DSS-Fn groups in experiment 2 (C); abundance changes of tryptophan, indole lactic acid, glycocholic acid and murideoxycholic acid in experiment 2 (the color changes represent the abundance changes of metabolites) (D).

**Figure S3.** Design of the minimal microbial community. Phylogenetic analysis of SynCom members (A); competition indces among SynCom members were calculated using the KEGG Orthology information of the annotated genomic data from each species (B).

**Figure S4.** Competition and complementarity relationships among SynCom members and Fn. Growth curves of SynCom1 (Data are mean ± SEM) (A), SynCom2 (Data are mean ± SEM) (B), SynCom3 (Data are mean ± SEM) (C) and SynCom4 (Data are mean ± SEM) (D) in GAM medium (*n* = 3 biologically independent experiments); growth curves of the SynCom in mGAM medium. Data are mean ± SEM. *n* = 3 biologically independent experiments (E); pH changes of SynCom in GAM, and pH changes of SynCom+Fn in GAM and mGAM. Data are mean ± SEM. *n* = 3 biologically independent experiments (F); pH changes of SynCom+Fn in GAM containing 1% arginine or GAM containing 1% tryptophan. Data are mean ± SEM. *n* = 3 biologically independent experiments (G); tryptophan metabolism in human fecal metabolomics (the color changes represent the abundance changes of metabolites) (H); the arginine metabolism in human fecal metabolomics (I); the tryptophan metabolism in mouse fecal metabolomics (J); the arginine metabolism in mouse fecal metabolomics (K); blood routine test. Data are mean ± SEM. **P* < 0.05, ***P* < 0.01 by Student’s t-test (L-M).

**Figure S5.** Blood TNF-α, IgA, IgD, IgE, IgE, IgG and IgM levels of different groups. Data are mean ± SEM. *n* = 6. ns > 0.05 by ANOVA test.

**Figure S6.** SynCom alleviates *F. nucleatum*-induced carcinogenesis. The most abundant 20 genera in different groups (A); differential species among MF, MF_LSyn and MF_HSyn groups. *n* = 6. Data are mean ± SEM. P values were calculated by Kruskal-Wallis test (B); the most abundant 20 KEGG pathways in different groups (C); differential KO annotations of metagenome data among MF, MF_LSyn and MF_HSyn groups. *n*=6. Data are mean ± SEM. P values were calculated by Kruskal-Wallis test (D); Procrustes analysis between species and KO annotations (E); the fungal community composition at the species level (F); GSEA enrichment analysis based on the metabolomics data between MF_HSyn and MF groups (NES, normalized enrichment score) (G); GSEA enrichment analysis based on the metabolomics data between MF_HSyn and MF_LSyn groups (H); the abundance of LPS 18:2, PS [18:1(9Z)/0:0], 5-hydroxyindole and palmitamide in MF and MF_LSyn groups. Data are mean ± SEM. *n* = 6. **P* < 0.05 by Student’s t-test (I); the abundance of gamma-glutamylleucine and FA 18:1+30 in MF and MF_HSyn groups. Data are mean ± SEM. *n* = 6. **P* < 0.05 by Student’s t-test (J).

**Fig. S1**





**Fig. S2**


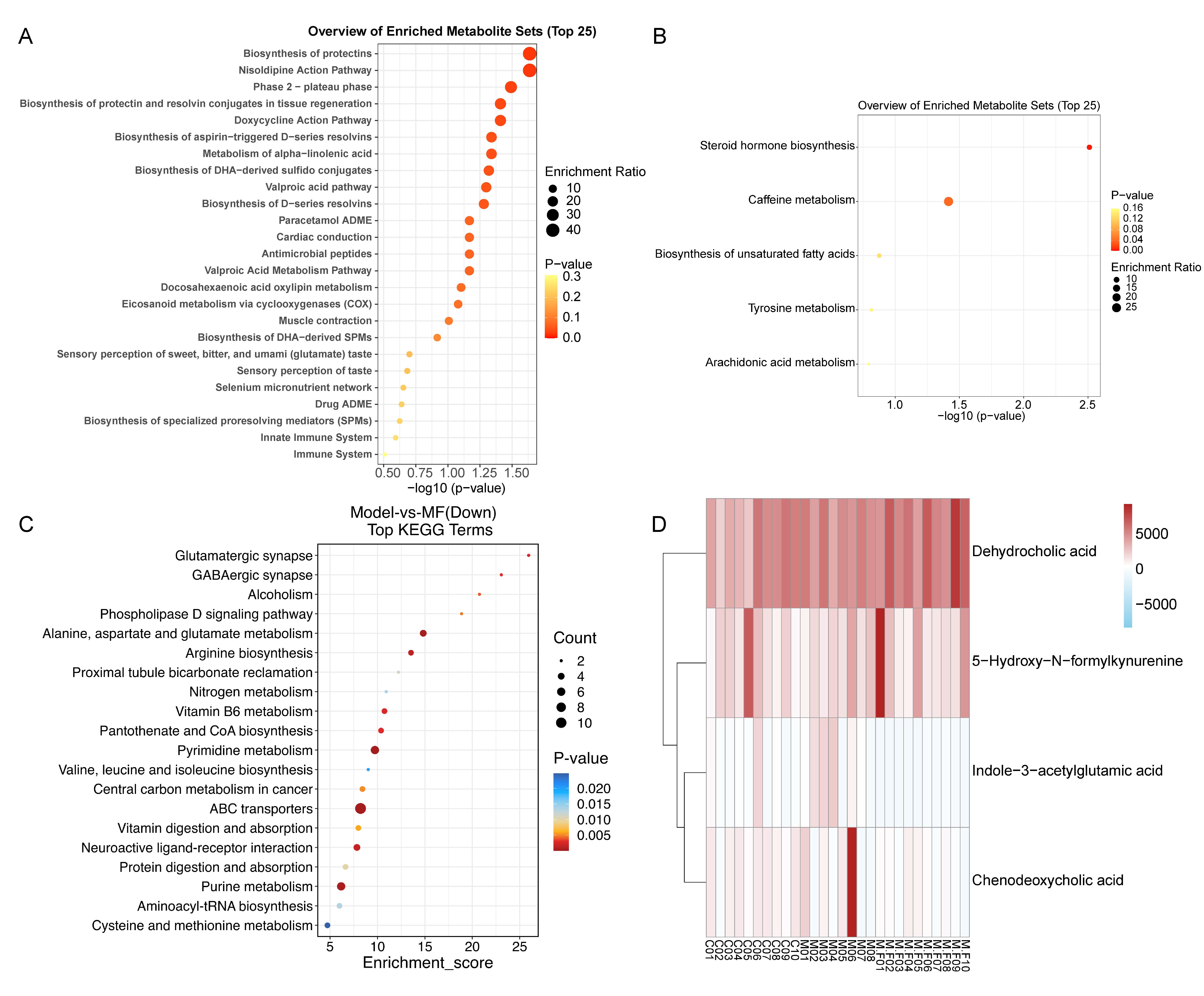


**Fig. S3**


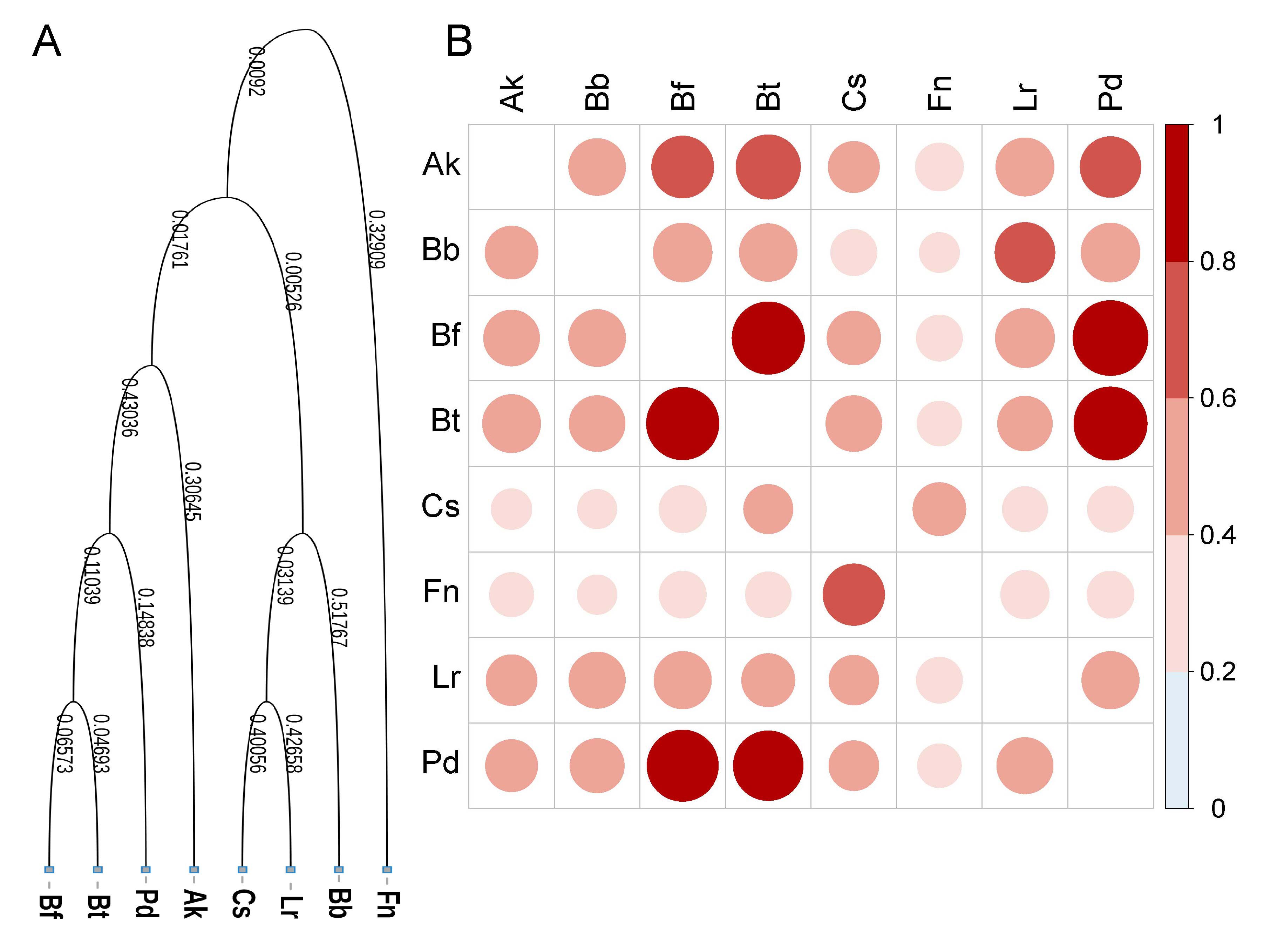


**Fig. S4**


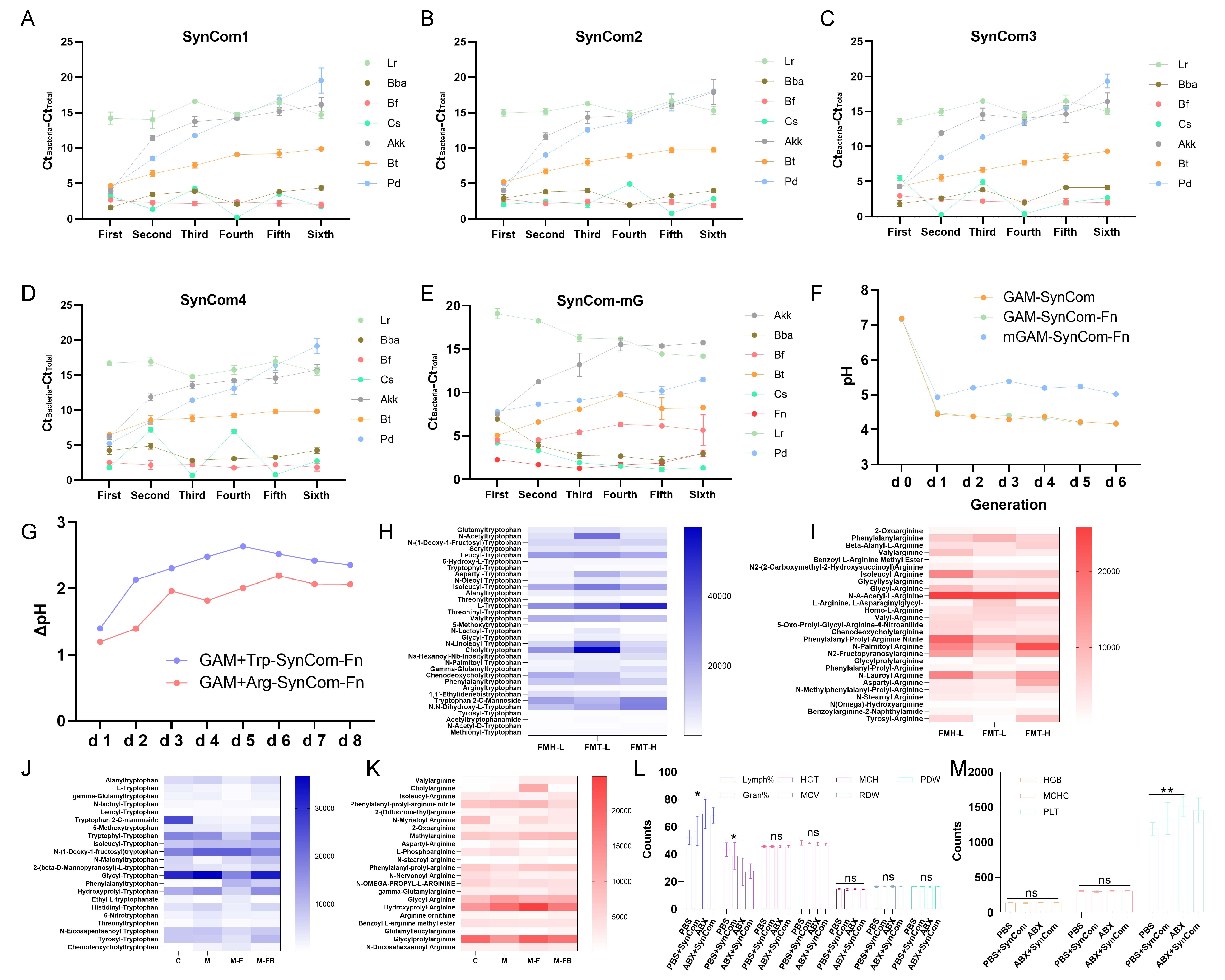


**Fig. S5**


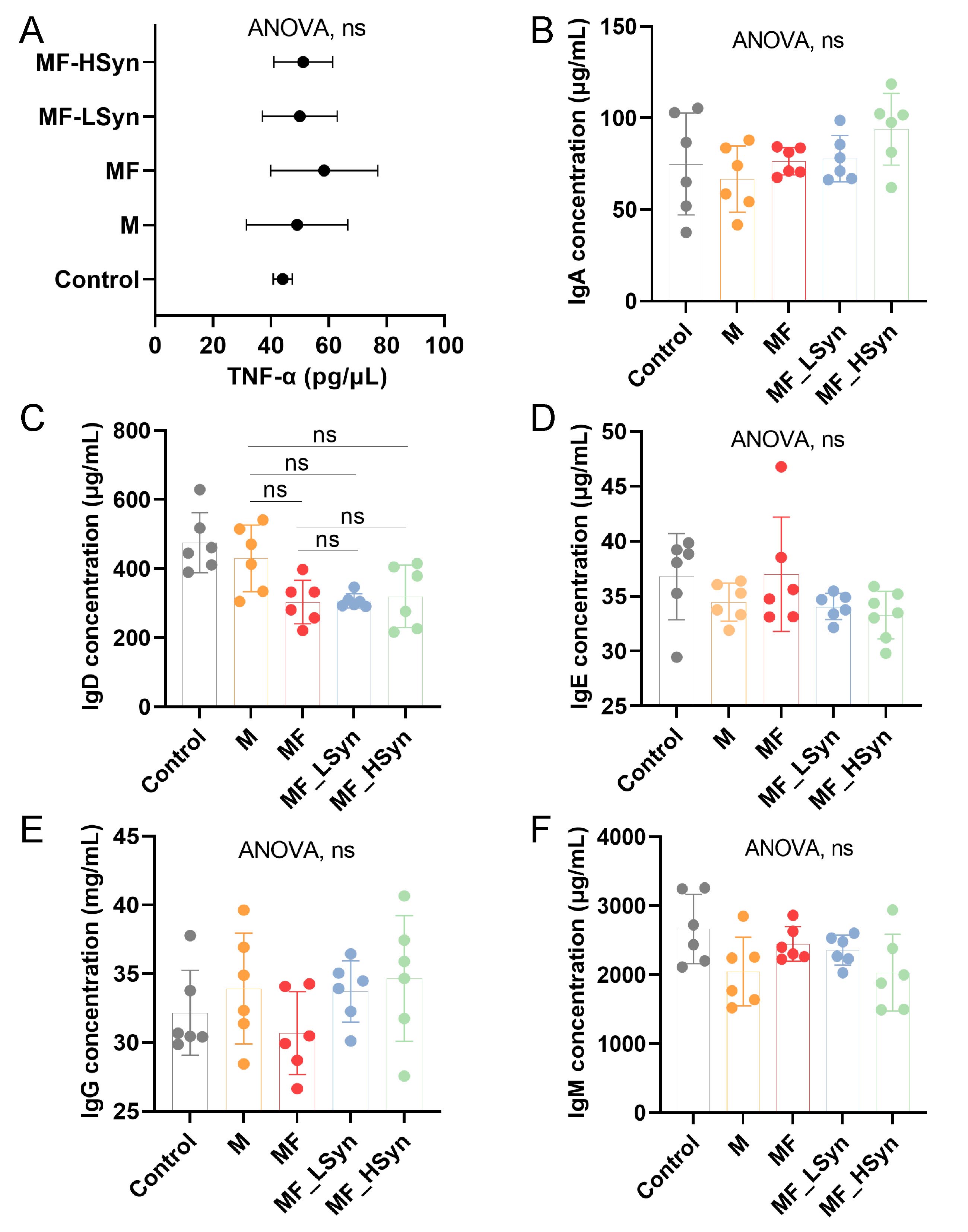


**Fig. S6**





References

[1] E. Pasolli, L. Schiffer, P. Manghi, A. Renson, V. Obenchain, D.T. Truong, F. Beghini, F. Malik, M. Ramos, J.B. Dowd, C. Huttenhower, M. Morgan, N. Segata, L. Waldron, *Nat. Methods.* **2017**, *14*, 1023-1024.

[2] A.D. Kostic, A.I. Ojesina, C.S. Pedamallu, J. Jung, R.G. Verhaak, G. Getz, M. Meyerson, *Nat. Biotechnol.* **2011**, *29*, 393-396.

[3] T. Seemann, *Bioinformatics* **2014**, *30*, 2068-2069.

[4] S. Kumar, G. Stecher, M. Li, C. Knyaz, K. Tamura, *Mol. Biol. Evol.* **2018**, *35*, 1547-1549.

[5] S. Xu, L. Li, X. Luo, M. Chen, W. Tang, L. Zhan, Z. Dai, T.T. Lam, Y. Guan, G. Yu, *Imeta* **2022**, *1*, e56.

[6] T. Wu, E. Hu, S. Xu, M. Chen, P. Guo, Z. Dai, T. Feng, L. Zhou, W. Tang, L. Zhan, X. Fu, S. Liu, X. Bo, G. Yu, *Innovation (Camb)* **2021**, *2*, 100141.

[7] S.Y. Shin, E.B. Fauman, A.K. Petersen, J. Krumsiek, R. Santos, J. Huang, M. Arnold, I. Erte, V. Forgetta, T.P. Yang, K. Walter, C. Menni, L. Chen, L. Vasquez, A.M. Valdes, C.L. Hyde, V. Wang, D. Ziemek, P. Roberts, L. Xi, E. Grundberg, M. Waldenberger, J.B. Richards, R.P. Mohney, M.V. Milburn, S.L. John, J. Trimmer, F.J. Theis, J.P. Overington, K. Suhre, M.J. Brosnan, C. Gieger, G. Kastenmüller, T.D. Spector, N. Soranzo, *Nat. Genet.* **2014**, *46*, 543-550.

[8] A. Belcour, C. Frioux, M. Aite, A. Bretaudeau, F. Hildebrand, A. Siegel, *Elife* **2020**, *9*, e61968.

[9] P.D. Karp, P.E. Midford, R. Billington, A. Kothari, M. Krummenacker, M. Latendresse, W.K. Ong, P. Subhraveti, R. Caspi, C. Fulcher, I.M. Keseler, S.M. Paley, *Brief. Bioinform.* **2021**, *22*, 109-126.

[10] Y. Cao, Y. Wang, X. Zheng, F. Li, X. Bo, *BMC Bioinformatics* **2016**, *17*, 294.

[11] C.A. Smith, E.J. Want, G. O'Maille, R. Abagyan, G. Siuzdak, *Anal. Chem.* **2006**, *78*, 779-787.

[12] S. Qu, L. Fan, Y. Qi, C. Xu, Y. Hu, S. Chen, W. Liu, W. Liu, J. Si, *Microbiol. Spectr.* **2021**, *9*, e0073021.

[13] D. Dodd, M.H. Spitzer, W. Van Treuren, B.D. Merrill, A.J. Hryckowian, S.K. Higginbottom, A. Le, T.M. Cowan, G.P. Nolan, M.A. Fischbach, J.L. Sonnenburg, *Nature* **2017**, *551*, 648-652.

[14] J. Papaparaskevas, V. Mela, D.P. Houhoula, A. Pantazatou, G.L. Petrikkos, A. Tsakris, *J. Clin. Microbiol.* **2013**, *51*, 1593-1595.

[15] J. Junick, M. Blaut, *Appl. Environ. Microbiol.* **2012**, *78*, 2613-2622.

[16] S.H. Wong, T.N. Kwong, T.-C. Chow, A.K. Luk, R.Z. Dai, G. Nakatsu, T.Y. Lam, L. Zhang, J.C. Wu, F.K. Chan, *Gut* **2017**, *66*, 1441-1448.

[17] Z. Zhou, Y. Wang, R. Ji, D. Zhang, C. Ma, W. Ma, Y. Ma, X. Jiang, K. Du, R. Zhang, P. Chen, *Front. Pharmacol.* **2022**, *4*, 841918.

[18] J.E. Wells, K.B. Williams, T.R. Whitehead, D.M. Heuman, P.B. Hylemon, *Clin. Chim. Acta.* **2003**, *331*, 127-134.

[19] B.H. Mullish, J.A.K. McDonald, A. Pechlivanis, J.R. Allegretti, D. Kao, G.F. Barker, D. Kapila, E.O. Petrof, S.A. Joyce, C.G.M. Gahan, I. Glegola-Madejska, H.R.T. Williams, E. Holmes, T.B. Clarke, M.R. Thursz, J.R. Marchesi, *Gut* **2019**, *68*, 1791-1800.

[20] J. She, G. Tuerhongjiang, M. Guo, J. Liu, X. Hao, L. Guo, N. Liu, W. Xi, T. Zheng, B. Du, B. Lou, X. Gao, X. Yuan, Y. Yu, Y. Zhang, F. Gao, X. Zhuo, Y. Xiong, X. Zhang, J. Yu, Z. Yuan, Y. Wu, *Cell. Metab.* **2024**, *36*, 408-421.e405.
